# Gut microbiome and plasma metabolome changes in rats after oral gavage of nanoparticles: sensitive indicators of possible adverse health effects

**DOI:** 10.1186/s12989-022-00459-w

**Published:** 2022-03-23

**Authors:** Robert Landsiedel, Daniela Hahn, Rainer Ossig, Sabrina Ritz, Lydia Sauer, Roland Buesen, Sascha Rehm, Wendel Wohlleben, Sibylle Groeters, Volker Strauss, Saskia Sperber, Haleluya Wami, Ulrich Dobrindt, Karola Prior, Dag Harmsen, Bennard van Ravenzwaay, Juergen Schnekenburger

**Affiliations:** 1 Experimental Toxicology and Ecology, BASF SE, 67056 Ludwigshafen am Rhein, Germany; 2 Institute of Pharmacy, Pharmacology and Toxicology, Freie Universität Berlin, 14195 Berlin, Germany; 3 Biomedical Technology Center of the Medical Faculty, University of Muenster, Mendelstrasse 17, 48149 Muenster, Germany; 4 HB Technologies AG, 72076 Tübingen, Germany; 5 Medical Data Integration Center, University Tuebingen, 72072 Tübingen, Germany; 6 Polymer Physics, BASF SE, 67056 Ludwigshafen am Rhein, Germany; 7 Institute of Hygiene, University of Muenster, 48149 Muenster, Germany; 8 Department of Periodontology and Operative Dentistry, University Hospital Muenster, 48149 Muenster, Germany

**Keywords:** Nanomaterial, Metabolomics, Gut microbiota, Intestinal microbiome, Oral nanoparticle administration, Silver nanoparticles, SiO_2_ nanoparticles

## Abstract

**Background:**

The oral uptake of nanoparticles is an important route of human exposure and requires solid models for hazard assessment. While the systemic availability is generally low, ingestion may not only affect gastrointestinal tissues but also intestinal microbes. The gut microbiota contributes essentially to human health, whereas gut microbial dysbiosis is known to promote several intestinal and extra-intestinal diseases. Gut microbiota-derived metabolites, which are found in the blood stream, serve as key molecular mediators of host metabolism and immunity.

**Results:**

Gut microbiota and the plasma metabolome were analyzed in male Wistar rats receiving either SiO_2_ (1000 mg/kg body weight/day) or Ag nanoparticles (100 mg/kg body weight/day) during a 28-day oral gavage study. Comprehensive clinical, histopathological and hematological examinations showed no signs of nanoparticle-induced toxicity. In contrast, the gut microbiota was affected by both nanoparticles, with significant alterations at all analyzed taxonomical levels. Treatments with each of the nanoparticles led to an increased abundance of Prevotellaceae, a family with gut species known to be correlated with intestinal inflammation. Only in Ag nanoparticle-exposed animals, *Akkermansia*, a genus known for its protective impact on the intestinal barrier was depleted to hardly detectable levels. In SiO_2_ nanoparticles-treated animals, several genera were significantly reduced, including probiotics such as *Enterococcus*. From the analysis of 231 plasma metabolites, we found 18 metabolites to be significantly altered in Ag-or SiO_2_ nanoparticles-treated rats. For most of these metabolites, an association with gut microbiota has been reported previously. Strikingly, both nanoparticle-treatments led to a significant reduction of gut microbiota-derived indole-3-acetic acid in plasma. This ligand of the arylhydrocarbon receptor is critical for regulating immunity, stem cell maintenance, cellular differentiation and xenobiotic-metabolizing enzymes.

**Conclusions:**

The combined profiling of intestinal microbiome and plasma metabolome may serve as an early and sensitive indicator of gut microbiome changes induced by orally administered nanoparticles; this will help to recognize potential adverse effects of these changes to the host.

**Supplementary Information:**

The online version contains supplementary material available at 10.1186/s12989-022-00459-w.

## Background

The oral exposure to nanoparticles (NP) requires solid models for the assessment of potential hazards. This includes systemic effects due to nanoparticles absorbed from the intestinal tract as well as local effects on the intestine and the gut microbiota. While the systemic availability of NP is often low [1], local effects on the intestine may be more relevant. Effects of foodborne NP on the gut microbiota are not well-understood, much less the consequences of these changes to the host organisms [2]. Food grade Silica SiO_2_ NP (E551) are mainly used as an anticaking food additive to support the flowability of powdered products. In addition, they are also used in cosmetics and in medical products. The average human dietary exposure of amorphous silica was estimated to be 9.4 mg/kg body weight/day of which 1.8 mg/kg body weight/day seems to be nano-sized [3]. Silver NP are added as antimicrobial additives in medical products (e.g. wound dressings, hand gels, cavity filler), food packaging and kitchenware, but also as pearlescent pigments in coatings of confectionary and chocolates (E 174) [4–6]. The commercialization of these nanoparticle-based products is expanding with a global production of up to 1.5 million tons of SiO_2_ NP and more than 500 tons annually for Ag NP, thus increasing the potential for oral uptake [7]. Safety assessment of these NP should therefore increasingly focus on oral toxicity studies.

Most of the available nanomaterial oral uptake studies have analyzed Ag NP in rodent models. Some studies observed Ag NP-induced loss of body weight, inflammatory responses, hepatotoxicity [8–10], and/or cardiotoxicity (for review see Bostan et al*.* 2016 [11]) whereas many others found no adverse health effects [12–17]. Notably, Hadrup et al*.* [18] observed toxic effects of ionic silver but not of equimolar Ag NP (9 mg/kg body weight/day) after oral gavage of Wistar rats for 28 days. Oral studies in rodents using SiO_2_ NP also described either signs of toxicity in different organs such as liver, lung and testis [3, 19], or no adverse effects depending on the specific NP [20–24]. Between 0.4 and 18% of orally administered silver has been described to be absorbed in mammals and distributed to different organs with the highest levels being observed in the intestine and stomach [25]. Fecal excretion rates were 98% for rats, 99.6% for mice, 90% for dogs and 98% for monkeys [26] indicating a low bioavailability in rodents, but also meaning that they have been in contact with the rodent gut microbiota. Detrimental Ag NP effects on the gut microbiota are conceivable due to known antimicrobial properties.

The gut microbiota of mammalians consists of approx. 10^14^ intestinal microbes belonging to more than 1000 different species. The gut microbiota is involved in a vast array of functions including energy utilization, drug metabolism and immunity. Changes in gut microbiota are associated with a broad variety of diseases and influence disease development and progression [27]. Recent work documented an association of minor alterations in gut microbial communities with gastro-intestinal diseases such as tumors and inflammations in humans [28]. The depletion of a single bacterial species, for instance, is associated with human IBD [29]. Even more intriguing, a gut-brain-axis has been proposed and data suggest that the gut microbiota also plays a role in the regulation of anxiety, mood, cognition, pain and stress [30–32]. Analyzing alterations in the gut microbiota is currently a major issue in diagnostics and in the evaluation of drug function and treatment [33–36]. The current research of gut microbiomes has identified several species with major impact on pathophysiological alterations, intestinal barrier integrity or inflammatory processes [37].

Despite its potential impact on human and animal health, gut microbiota has not yet been in the focus of research in the field of nanotoxicology. To date, only limited information is available regarding the effects of NP on gut microbiota, although alterations of the microbial community structure may be utilized as an important endpoint for nanotoxicology [2]. A few studies investigating the influence of Ag NP on intestinal microbes have been published so far [2, 38]. Most of these studies used in vitro cultures of gut microbiota, which provided only limited information, since numerous intestinal bacterial species cannot be cultured under standard in vitro conditions. Important data gaps exist in the comparison of clinical and pathological findings in the animals orally exposed to NP and the identification of altered bacterial species with known functions in the homeostasis of the host organism’s health state. Moreover, the effectors of microbiome changes in nanotoxicology are still unknown.

Recent work has demonstrated a large impact of the gut microbiota on mammalian blood metabolites, suggesting a major interplay between bacterial and mammalian metabolism and providing mechanistic insights into the function of the microbiome for the host organism [39–41]. Intestinal microbes produce different vitamins and metabolites such as short-chain fatty acids, thereby providing nutritional support for the host [42]. It has been estimated that 10% of the metabolites found in mammalian blood are derived from the gut microbiota [39]. These metabolites are being increasingly recognized as key molecular mediators of microbiome influence on disease and as an essential part of host physiology with multiple effects on immune function and intestinal homeostasis [41, 43, 44].

Metabolomics allows the indirect study of gut microbiome effects in easily accessible body matrices like urine, blood or feces. Previous studies identified possible modes of action or adverse outcomes after substance treatment using plasma metabolomics in rats [45]. Substances, which produce toxic effects via a common mode of action produced a set of common metabolite changes. Consistently regulated metabolites can therefore be used to establish toxicity related metabolic patterns as an indicator of drug or chemical induced intestinal microbial community changes [46–48].

Here, we present data from a subacute oral toxicity study in accordance with OECD test guideline no. 407 with a daily administration of 1000 mg/kg body weight of SiO_2_ NP or 100 mg/kg body weight of Ag NP to male Wistar rats. We comprehensively assessed potential toxic effects of these nanoparticles based on clinical parameters and analyzed composition and abundance of the gut microbiome community. To address effectors of the microbial alterations, we additionally analyzed the levels of metabolites in the plasma of SiO_2_ and Ag NP-treated animals by liquid- and gas-chromatography coupled to mass-spectrometry.

## Results

The effect of ingested NP on classic toxicological in vivo endpoints and on the gut microbiome and microbiome-affected plasma metabolome was assessed by a 28-day oral uptake study in male Wistar rats following the OECD test guideline no. 407 (TG 407). Here, we performed a limit dose test, which is the preferred test when toxicity is expected to be low and lethality is unlikely at the limit dose. We used the suggested dose level of 1000 mg/kg body weight/day for SiO_2_ NP [49]. For Ag NP, with an expected higher toxic potential, 100 mg/kg body weight/day was used. The applied dose is well below the LD_50_ of 280 mg/kg body weight/day found for ionic silver in rats [25, 50], and therefore it seemed to be appropriate to follow TG 407.

### Neither Ag NP nor SiO_2_ NP induced adverse effects detected by clinical observations or pathology

#### In-life data of Ag NP-treated animals: clinical examination, food and water consumption, body weight development

All animals treated with Ag50 EO (herein referred to as Ag NP) showed black-discolored feces. The effect was assessed as being related to the test substance but not as being adverse. Food- and water-consumption of all animals were not affected, and the body weight development was not impaired (Additional file 1: Table S1).

#### Clinical pathology: hematology, clinical chemistry, acute phase proteins, urine analysis

Nearly all parameters measured in blood and urine samples of Ag NP-treated animals did not differ significantly from the values of the control animals. The following significant changes were observed but assessed not to be related to NP-treatment (see Table 1 and Additional file 1: Tables S2–S4).

**Table 1:** Overview of findings per test groups upon 28-day oral gavage administration of the respective nanomaterials

Parameter	Control group	SiO_2_		Ag	
Body weight: day 28 (g)	Mean: 286.2 SD: 27.5	Mean: 294.5 SD: 18.1		Mean: 296.1 SD: 21.8	
			*p* value: 0.5868^a^		*p* value: 0.5451^a^
Hematology: RBC (tera/L)	Mean: 7.94 SD: 0.24	Mean: 8.27 SD: 0.46		Mean: 8.4 SD: 0.21	
			*p* value: 0.2222^b^		*p* value: 0.0317^b^
Hematology: RET (%)	Mean: 2.2 SD: 0.3	Mean: 2.1 SD: 0.3		Mean: 1.7 SD: 0.3	
			*p* value: 0.8413^b^		*p* value: 0.0397^b^
Hematology: MCHC (mmol/L)	Mean: 20.58 SD: 0.07	Mean: 20.81 SD:0.38		Mean: 20.41 SD: 0.12	
			*p* value: 0.2222^b^		*p* value: 0.0317^b^
Hematology: Eos. (%)	Mean: 1.5 SD: 0.5	Mean: 1 SD: 0.3		Mean: 1.7 SD: 0.5	
			*p* value: 0.0238^b^		*p* value: 0.4127^b^
Clinical chemistry: Hapt. (ng/mL)	Mean: 234.02 SD: 103.09	Mean: 413.56 SD: 198.9		Mean: 287.83 SD: 179.12	
			*p* value: 0.0476^c^		*p* value: 0.4206^c^
Urine-analysis	Various data sets	Not sign. different from control		Not sign. different from control	
Relative liver weights (g)	Mean: 2.445 SD: 0.108	Mean: 2.706 SD: 0.117		Mean: 2.586 SD: 0.142	
			*p* value: 0.0159^b^		*p* value: 0.1508^b^
Relative organ weights	Various data sets	Not sign. different from control		Not sign. different from control	
Gross lesions				Discolouration of the content of the glandular stomach, jejunum, cecum, and/or colon	
Histopathology	Various data sets	Not sign. different from control		Not sign. different from control	

SiO_2_: 1000 mg/kg body weight/day SiO_2_ NP; Ag: 100 mg/kg body weight/day Ag NP, assessed as incidental or test substance-related (see Additional file 1: Tables S2–S5 for details on the respective findings and Buesen et al*.* 2014 for SiO_2_ data [51]). *RBC* red blood cells, *MCHC* mean corpuscular hemoglobin concentration, *WBC* white blood cells, *Eos* eosinophils, *Hapt* haptoglobin. N = 5

^a^Student *t*-test (two sided)

^b^Wilcoxon test (two-sided)

^c^Wilcoxon test (one-sided)

In animals treated with Ag NP, red blood cell (RBC) counts were higher (8.4 Tera/L) and mean corpuscular hemoglobin concentration (MCHC; 20.41 mmol/L) as well as relative reticulocyte counts (1.7%) were lower compared to control animals. However, RBC and relative reticulocyte counts were within historical control ranges (RBC: 7.59–8.60 Tera/L, relative reticulocyte counts: 1.4–3.1%). The calculated MCHC value was slightly below the historical control range (MCHC: 20.43–23.73 mmol/L), but all measured red blood cell parameters in these individuals were either within the study control range (hematokrit and hemoglobin) or the historical control range (RBC) and were therefore regarded as incidental and not treatment-related.

#### Pathology: gross lesions, absolute and relative organ weights, histopathology

In various locations of the digestive tract, a discoloration of the content was observed in Ag NP-treated animals. This discoloration was regarded to be caused by the test substance. We did not recognize discoloration of any tissue, i.e. of the mucosa of the organs of the gastrointestinal tract. We do not assume that this is a sign of argyria as observed for human skin [25]. Most likely, the presence of the silver particles in the lumen of the gastrointestinal tract themselves changed the color of its content. Macroscopically, no change of the color of the mucosa was noticed. Small particles of yellowish to dark color, however, were observed during microscopic examinations of the duodenum, i.e. in the submucosa of the villi tip and within macrophages.

In addition, all animals showed minimal to slight inflammatory cell infiltrates in the submucosa of the glandular stomach. No other treatment-related macroscopic and histopathological findings were observed. No treatment-related organ weight changes were detected in any of the animals (data not shown).

#### In-life data of SiO_2_ NP-treated animals

The clinical pathology results for SiO_2__naked (herein referred to as SiO_2_ NP) had already been published by Buesen et al*.* [51]. No treatment-related changes regarding in life data, clinical pathology or histopathology had been reported. Data shown in Tables S1–S4 (Additional file 1) are provided only for comparison, for further details see Buesen et al*.* [51].

### Gut microbiota profiling at the onset of gavage of male Wistar rats

To determine if the oral exposure of nanoparticles affects the rat intestinal microbes, we performed a 16S rRNA microbial/taxonomic profiling of different feces samples. First, feces were collected from each animal one day before the beginning of the treatment (untreated control, UC). Second, feces were collected at day 25 after the daily gavage with either PBS + BSA (vehicle control group, VC), Ag NP (100 mg/kg body weight/day) or SiO_2_ NP (1000 mg/kg body weight/day). DNA from these samples was extracted followed by next generation sequencing (NGS) analysis and DNA sequences were analyzed with QIIME2 using the SILVA database.

The total observed number and relative abundance of amplicon sequence variants (ASVs) defined by α-diversity did not differ significantly between the treatment groups and untreated controls, as illustrated by the Shannon–Wiener index or the Inverse Simpson index (Additional file 1: Fig. S1). Similar results have been reported by Wilding et al*.* [52] after a 28-day administration of Ag NP to mice. To compare the diversity of gut microbiota between the samples and to assess the level of differentiation between the treatment groups, β-diversity was calculated based on ASV information of all samples and visualized using Principal Coordinates Analysis (PCoA). The untreated controls (UCs) formed a cluster and were separated from all other samples collected after treatment (Additional file 1: Fig. S2a). The individual samples at day 25 of the study were also more separated from each other, compared to samples of same rats before treatment (day 0). The common distance of the UC group to all samples after 25 days of gavage most likely reflects primarily the time-dependent turnover that all microbial communities underwent during the progress of the 25-day observation period, impacted by sexual maturation and further developmental changes during aging of the rats. In addition, the procedure of gavage may have induced stress, not notable by the clinical observations, and other factors able to influence the balance of gut microbial composition. The abundance of phyla and classes was further assessed by taxonomic assignment of the ASVs and a comparison between untreated and vehicle-treated controls is shown in supplementary information (Additional file 1: Text section and Fig. S3, Tables S5, S6). More important, the tight clustering of the untreated control samples in the diversity analysis suggests that the individual rats were initially (at day 0) closely related and rather similar to each other with respect to their bacterial community structure. These relatively small differences in diversity between all rats confirm an appropriate baseline situation at the beginning of the treatments.

The comparison of β-diversity between the treatment groups at day 25 was then performed in a separate analysis including only the samples after the gavage period (Additional file 1: Fig. S2b). The samples belonging to individual treatment groups, and in particular the vehicle controls, appeared to be less aggregated compared to the tight clustering of samples at day 0. Nevertheless, a clear separation between samples of the different treatment groups (VC, Ag NP, SiO_2_ NP) could be observed, although some limited overlapping between vehicle control samples and the Ag NP group was noted. All samples after SiO_2_ NP treatment were completely separated from VCs and from samples after gavage with Ag, indicating that the individual rats had been developed into differentiated treatment groups, each with common characteristics of their gut microbiota.

### Ag NP and SiO_2_ NP induce distinct alterations in the gut microbiota of male Wistar rats

#### Effects of Ag NP

**Fig. 1:**
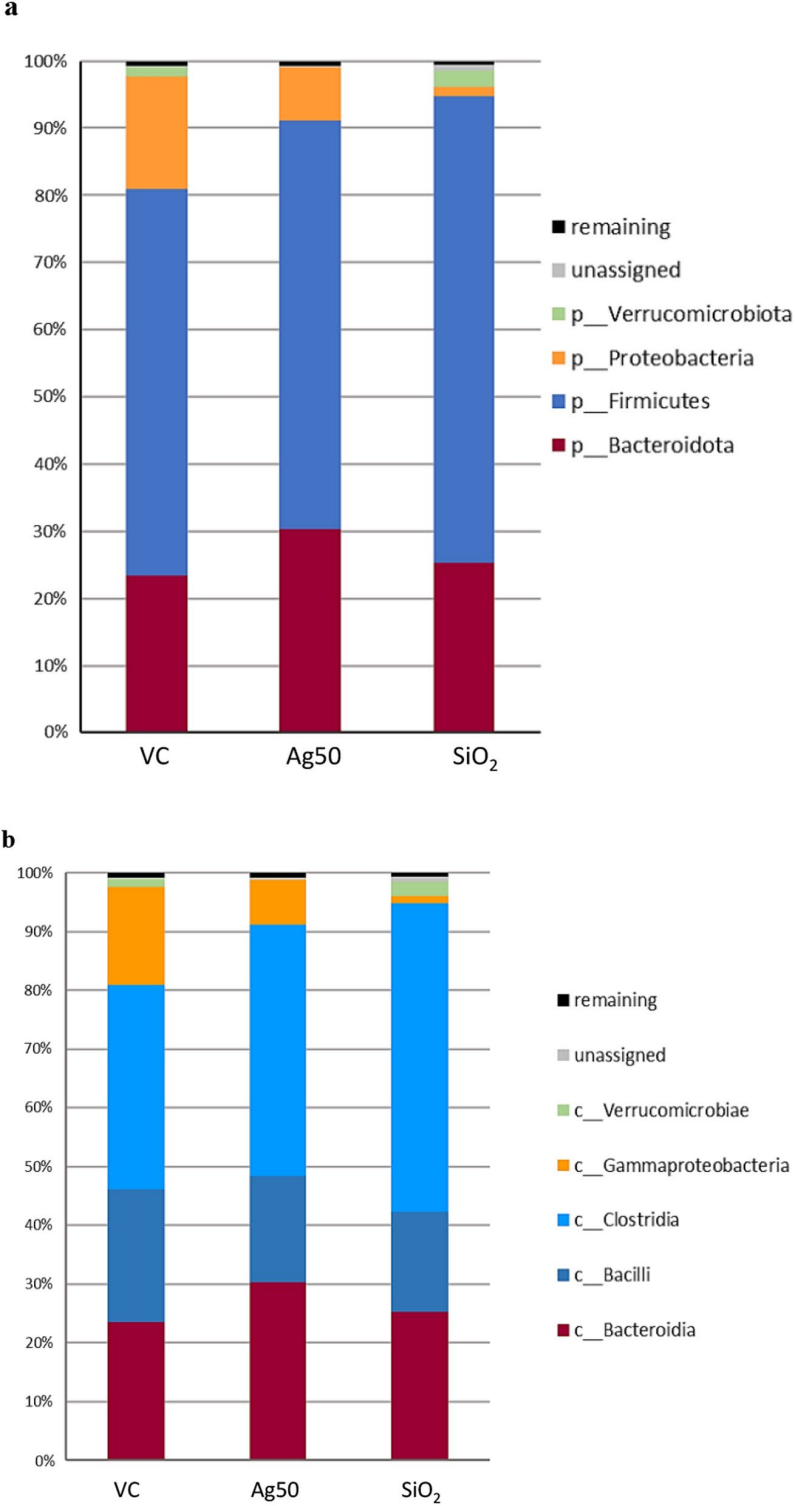

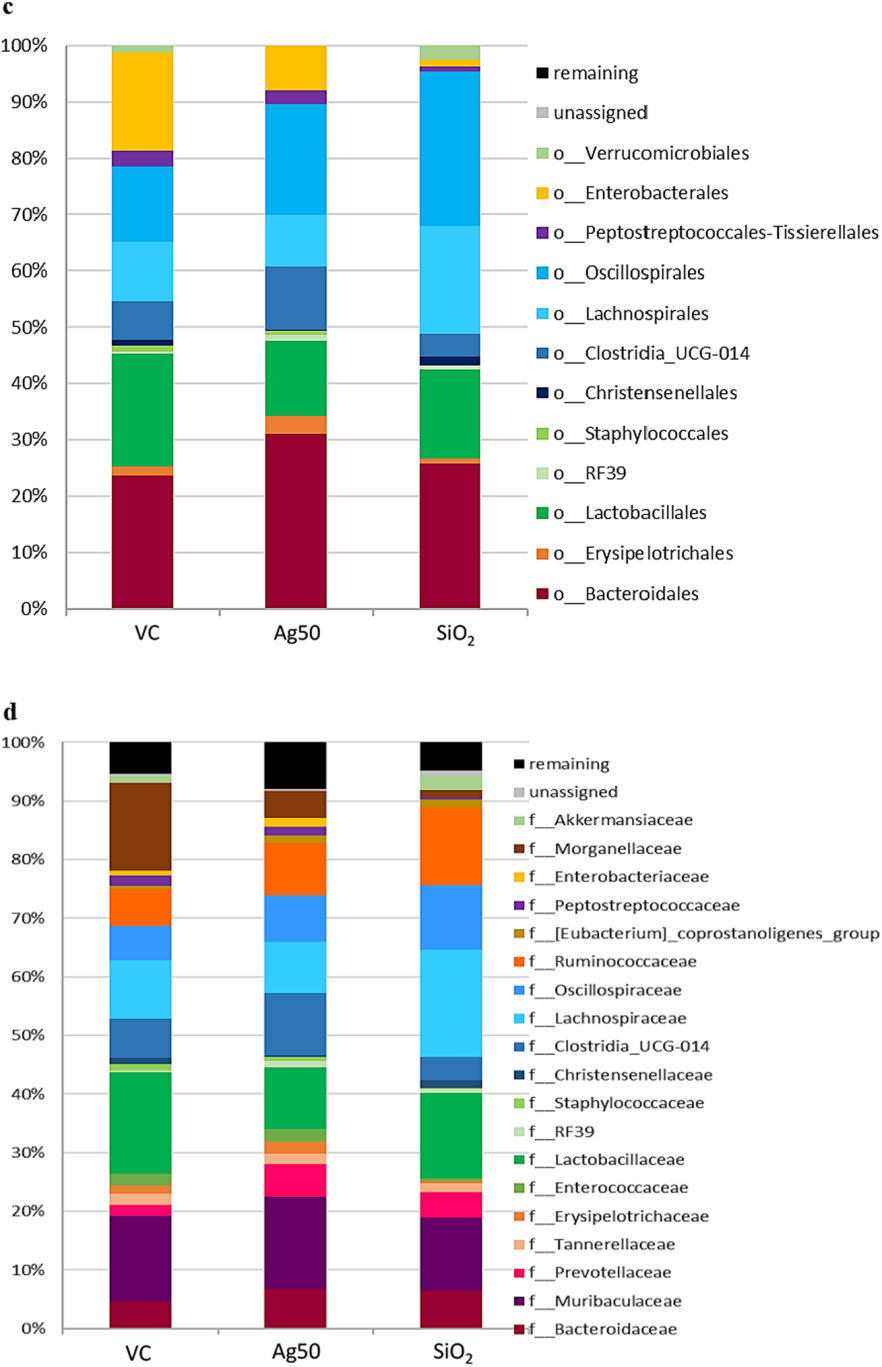
Relative abundance of bacterial phyla (**a**), classes (**b**), order (**c**) and family (**d**) in the gut microbiota of male Wistar rats after exposure to vehicle control, Ag or SiO_2_ nanoparticles. VC, Vehicle control (nanoparticle-free PBS + BSA); Ag50, Ag nanoparticles (100 mg/kg body weight/day); SiO_2_, SiO_2_ nanoparticles (1000 mg/kg body weight/day). Mean values of the relative abundance of the different ASVs from gut microbiota after a 25-day oral gavage, analyzed from feces samples of five animals in each group (N = 5). Values with abundance ≥ 1% in at least one group are shown for each taxonomic level

To assess effects of Ag NP on the rat intestinal microbiome, we analyzed the ASVs after taxonomic assignment from level 2 (phylum) to 6 (genus). As expected from the results of the β-diversity analysis, the inter-animal variability was relatively high in many cases. The enhanced variability likely arises from individual differences during the development and sexual maturation of the rats within the 25-day period of treatment, compared to the relatively small differences in diversity determined at day 0 (for further details see Additional file 1: Fig. S2a). Similar individual variabilities in rodent gut microbiota have been reported before [53]. As shown in Fig. 1a, gavage of Ag NP (at 100 mg/kg body weight/day) led to structural changes in the gut microbiota at the level of phyla. In total, four different predominant phyla were identified with a relative proportion of more than 1%. The two most abundant phyla, Firmicutes and Bacteroidota, together represented about 81% of the total ASVs in vehicle treated animals, and more than 91% in rats after NP treatments. After gavage with Ag NP, the level of Bacteroidota increased from 23.5% in the controls (VC) towards 30.4% (*p* = 0.087), whereas Firmicutes stayed nearly at the same level (60.8% vs*.* 57.5% in VC, Fig. 1a, for further details see Additional file 1: Table S7). This led to a decrease of the Firmicutes/Bacteroidetes ratio (2:1 vs. 2.5:1 in VC). In addition, the phylum Verrucomicrobia was reduced in abundance from 1.26% (VC) to 0.04%, and the level of Proteobacteria decreased from 16.8% (VC) to 7.8% (changes not significant; Fig. 1a, Additional file 1: Table S7).

**Fig. 2:**
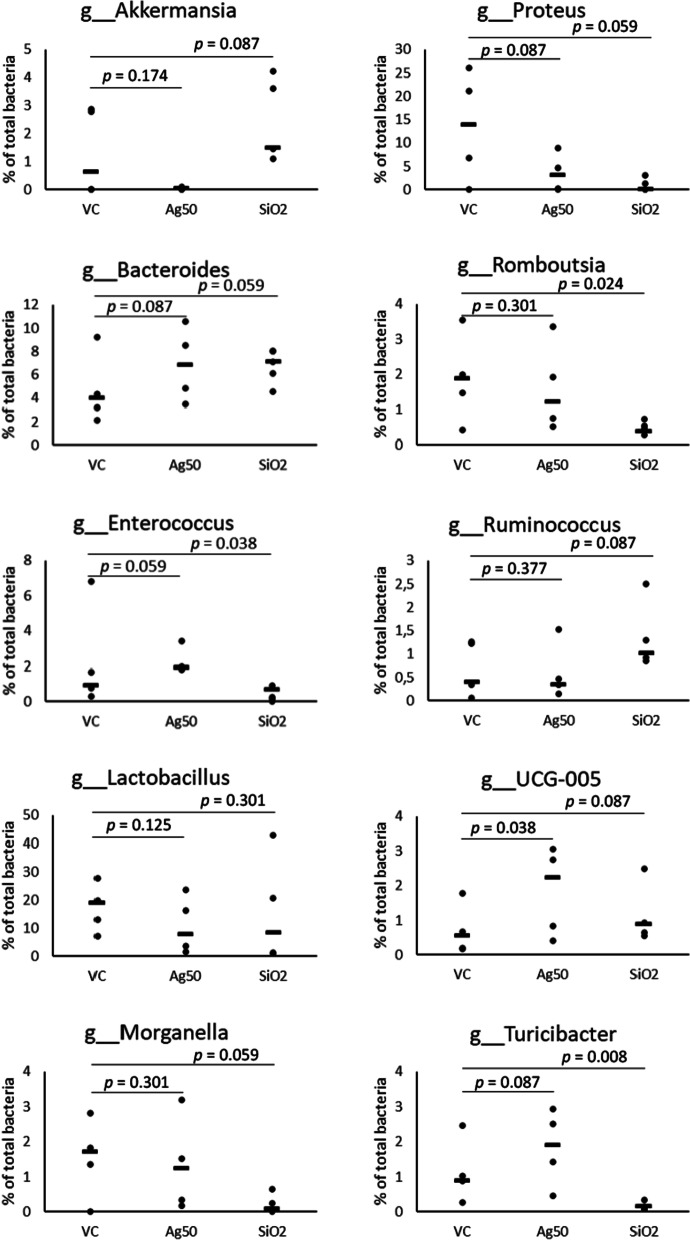
Scatter plots obtained for selected most abundant genera after exposure either to Ag or to SiO_2_. VC, vehicle control; Ag50, Ag nanoparticles (100 mg/kg body weight/day); SiO_2_, SiO_2_ nanoparticles (1000 mg/kg body weight /day); The median relative abundance of the selected genera displayed was ≥ 0.2% in at least one group (further abundant genera are shown in Fig. S4 (Additional file 1)*. p* values were obtained by Mann–Whitney-U-test. N = 5 for each group

Since both, beneficial and detrimental microbes are represented within these phyla, we analyzed the gut microbiota composition additionally at the level of class, order, family and genus to assess the effects of the Ag treatment in further detail (Fig. 1, Additional file 1: Table S8–S12). For the phylum Verrucomicrobia, we found the same distinct decrease to 0.04% in the subordinated family Verrrucomicrobiales and in genus *Akkermansia*, since this is the only identified genus representative for this phylum. A single species of this genus, *A. muciniphila,* usually accounts for 1–4% of the total gut microbiome and lives within the intestinal mucus layer in close proximity to the intestinal epithelial cells [54]. Accordingly, for our animal test groups, we determined a mean level of 1–4% of *Akkermansia*, except for Ag NP-treated animals, where *Akkermansia* was found to be virtually absent (Fig. 2). However, the five animals of the vehicle control displayed a relatively high variability in abundance of *Akkermansia* (Fig. 2) and thus, our data are not statistically significant.

The reduction in the phylum Proteobacteria seems to be mainly determined by a substantial decrease in abundance of the family Morganellacea (4.7% vs*.* 15.1% in controls, *p* = 0.087; Fig. 1d, Additional file 1: Table S10) and the genus *Proteus*, the third most abundant genus in control rats (3.4% vs. 13.5% in controls, *p* = 0.087; Fig. 2, Additional file 1: Table S11, S12). Whereas species in the genus *Proteus* are known to be opportunistic pathogens capable of causing major infections and disease problems in humans (e.g. urinary tract infections), their role in animals is still unclear, in some cases it may be symbiotic or change from neutral/commensal to parasitic [55].

In the nanoparticle-free control, the phylum Firmicutes is mainly composed of the classes Clostridia (34.9%) and Bacilli (22.6%). After gavage with Ag, we observed an increase in abundance of the class Clostridia and a reduction of members of the class Bacilli (42.8% and 18.0%, respectively, not significant), (Fig. 1b, Additional file 1: Table S8). The opposing trends seem to be mediated considerably by a decrease of Lactobacillales, which resides within the three most abundant orders identified (19.5% in controls vs. 12.8% in Ag-treated animals, *p* = 0.087), contrasting with the significant increase of Oscillospirales (18.9% vs. 13.2% in VC, *p* = 0.005) and Clostridia UCG-014 (10.7% vs. 6.7% in VC, *p* = 0.087; Fig. 1c, Additional file 1: Table S9). Notably, Erysipelotrichales, a rather low abundant order, which also belongs to Bacilli significantly exhibited a two-fold increase in abundance in Ag-treated rats (3.0% vs. 1.5% in VC, *p* = 0.008; Fig. 1c, Additional file 1: Table S9).

The changes in the order Lactobacillales further originated from a reduction of genus *Lactobacillus* (10.5% vs. 17.4% in VC, not significant; Fig. 2, Additional file 1: S11, S12), which was the most abundant genus in control rats. The observed elevation of the order Oscillospirales (Fig. 1c) was mainly determined by a significant increase of the family Ruminococcaceae (9.0% vs*.* 6.4% in VC; *p* = 0.038) and Oscillospiraceae (7.9% vs. 5.8% in VC, not significant; Fig. 1d, Additional file 1: Table S10). The associated genera responsible for the significant increase in abundance of the two families, Prevotellaceae (5.6% vs. 1.9% in the controls, *p* = 0.024) and Ruminococcaceae could not be identified in this analysis.

#### Effects of SiO_2_ NP

The results from rats orally exposed to 1000 mg/kg body weight/day SiO_2_ NP compared to those treated with vehicle control disclosed that SiO_2_ treatment also induced several structural alterations in the gut microbiota. At the level of phyla, we found a significant increase in Firmicutes (69.6% vs. 57.5% in controls, *p* = 0.008), whereas virtually no change was observed in the abundance of Bacteroidota (25.2% vs. 23.5% in VC) (Fig. 1a, Additional file 1: Table S7). Opposing to the effect determined for Ag, this rearrangement led to a slight increase in the Firmicutes/Bacteroidetes ratio (2.8:1 vs. 2.5:1 in controls). The alterations determined for the phylum Verrucomicrobia also differed clearly from those induced by Ag (Fig. 1a). The abundance of Verrucomicrobia and the subordinated genus *Akkermansia* raised to 2.4% after the gavage of SiO_2_ (vs. 1.3% in VC, *p* = 0.087).

Similar to the effects observed after Ag treatment, the phylum Proteobacteria decreased substantially in abundance from 16.8% in the controls to only 1.3% after SiO_2_ NP treatment (*p* = 0.038). As observed after treatment with Ag, we found a stringent reduction of the otherwise high abundant family Morganellaceae (1.1% vs*.* 15.1% in controls, *p* = 0.059; Fig. 1d, Additional file 1: Table S10) and of the genus *Proteus* (0.9% vs. 13.5% in controls, *p* = 0.059; Fig. 2, Additional file 1: Table S11, S12), to be mainly responsible for the reduction of the super ordinated phylum Proteobacteria.

Addressing the classes belonging to Firmicutes, we observed a significantly elevated level for Clostridia (52.5% vs*.* 34.9% in VC, *p* = 0.038) accompanied by a moderate decrease of the class Bacilli after SiO_2_ NP treatment (17.0% vs. 22.6% in VC, not significant) (Fig. 1b, Additional file 1: Table S8). The high abundant family Lactobacillaceae and the genus *Lactobacillus* (14.9% in SiO_2_ NP-treated vs. 17.4% in VC, not significant) mainly represented the class Bacilli. A significant reduction was determined for the family Staphylococcaceae and for the genus *Staphylococcus* (0.02% vs. 1.1% in VC, *p* = 0.014). Similarly, the family Enterococcaceae and the genus *Enterococcus* (0.4% vs. 2.1% in controls, *p* = 0.038; Fig. 2, Additional file 1: Tables S11, S12) were significantly reduced in rats after gavage with SiO_2_ NP.

Opposed to the changes after Ag treatment, we found a significant reduction for the genus *Turicibacter* (0.15% vs. 1.1% in controls, *p* = 0.008; Fig. 2, Additional file 1: Tables S11, S12) in SiO_2_-treated animals, contributing to a significant decrease in abundance of Erysipelotrichaceae (0.3% vs. 1.4% in controls,) and Erysipelotrichales (0.7% vs. 1.5% in controls; Fig. 1c, Additional file 1: Table S9, S10). We also observed a significant reduction in abundance of the order Peptostreptococcales-Tissierellales (0.9% vs. 2.8% in VC, *p* = 0.005) and of the genus *Romboutsia* in SiO_2_-treated rats (0.5% vs. 1.9% in controls, *p* = 0.024, Fig. 1c, Fig. 2, Additional file 1: Table S9, S11, 12).

Similar to the findings after oral treatment with Ag, the significant increase in Clostridia observed in SiO_2_-treated rats was mediated by a significant elevation of the two families Oscillospiraceae (11.2% vs. 5.8% in controls, *p* = 0.038) and Ruminococcaceae (13.4% vs*.* 6.4% in controls, *p* = 0.008; Fig. 1d, Additional file 1: Table S10). Genera accounting for these shifts could not be clearly identified. Other alterations within the class Clostridia differed from those observed for Ag treatment. In rats treated with SiO_2_ NP, a clear increase in the order Lachnospirales and the family Lachnospiraceae (18.5% vs*.* 10.1% in control, *p* = 0.038) was observed (Fig. 1c, 1d, Additional file 1: Table S9, S10). We also found the level of Prevotellacea being significantly increased after SiO_2_ NP exposure (4.5% vs. 1.9% in controls, *p* = 0.014; Fig. 1d, Additional file 1: Table S10). Similar increases in abundance of the family Prevotellacea were shown for Ag treatment, however, our analysis could not identify the associated genera.

### Ag NP and SiO_2_ NP led to changes in the level of specific metabolites in rat plasma

A potential impact of the gut microbiome on the host’s health and the prevention or progression of diseases is mediated most likely by microbiota-derived small molecules. The detection and analysis of these molecules within metabolome studies is therefore crucial for understanding microbiome effects. A summary of qualitative changes in the plasma metabolome of rats after the 28-day oral exposure of SiO_2_ NP has been published before by Buesen et al. [51]. Here, we analyzed the metabolomic changes in male rats orally exposed to Ag NP and SiO_2_ NP in more detail, including effects of the microbiota on the metabolome. We also compared the metabolome changes induced by the nanoparticles with the metabolome profiles of eight previously investigated antibiotics [40, 48] (Additional file 1: Table S13).

A total number of 231 metabolites were identified from blood plasma samples of treated rats. A summary of the significant changes observed in 18 different metabolites after exposure either to SiO_2_ NP or to Ag NP, compared to the vehicle-treated controls is shown in Table 2.

**Table 2:**
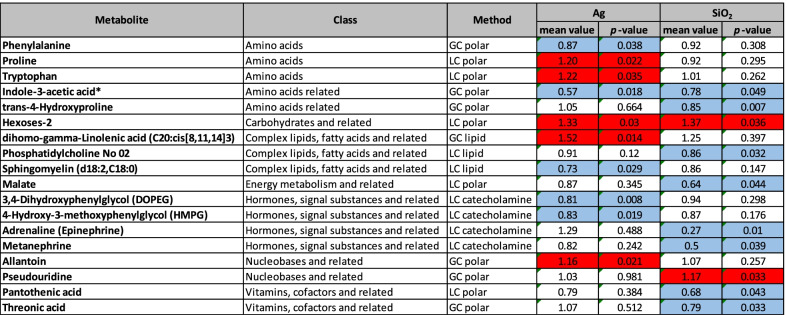
Significantly increased (red) or decreased (blue) metabolite levels in rat plasma

SiO_2_ nanoparticles: 1000 mg/kg body weight/day or Ag nanoparticles: 100 mg/kg body weight/day, mean values relative to vehicle controls (N = 5 per group, Welch t test; *p* < 0.05)

*Indicates gut microbiota-dependent metabolite

Each treatment group displayed significant changes in ten different plasma metabolites. In rats exposed to SiO_2_ NP, we identified two metabolites exhibiting elevated levels and eight metabolites with decreased levels, compared to the vehicle controls. After Ag NP treatment, a number of five different metabolites was found for each, increased and decreased level, respectively. Most of these effects were specific for the exposure to either Ag or SiO_2_. Metabolites with reverse response to Ag NP and SiO_2_ NP were not detected.

In the class of amino acids, we identified significant changes only in Ag NP-treated rats, with phenylalanine plasma levels being significantly reduced (0.87-fold compared to the controls) and proline as well as tryptophan being significantly increased (1.2- and 1.22-fold, respectively). In contrast, in the class of vitamins and related metabolites as well as in the class of energy metabolism and related we found significantly affected metabolites only in rats after SiO_2_ NP treatment. Pantothenic acid (vitamin B_5_) was 0.68-fold reduced, the vitamin C metabolite threonic acid displayed a 0.79-fold and malate a 0.64-fold reduction compared to the controls (Table 2).

Other metabolite classes were affected by both, SiO_2_ and Ag NP. Within the class of hormones, signal substances and related molecules, for instance, we found metabolites with decreased levels for either one of the treatments, respectively. In Ag NP-treated rats, DOPEG and HMPG were significantly reduced (0.81- and 0.83-fold of control levels, respectively), whereas in rats treated with SiO_2_ NP, epinephrine and metanephrine were significantly reduced (0.27 and 0.5-fold of control levels, Table 2). In contrast, in the class of nucleobases and related, we found metabolites with significantly increased levels for each treatment. Allantoin was increased 1.16-fold in Ag NP-treated rats, pseudouridine 1.17-fold in rats after SiO_2_ NP gavage (Table 2). In the class of carbohydrates and related, both nanoparticles seem to induce the level of hexose-2 to a similar extent (1.33-fold in Ag NP- and 1.37-fold in SiO_2_-treated rats, Table 2).

In the class of amino acids and related, SiO_2_-treated rats display significantly reduced levels of trans-4-hydroxyproline (0.85-fold compared to the controls). Most importantly, we found gut microbiota-derived indole-3-acetic acid (IAA) to be significantly reduced in the plasma of both, Ag NP- (0.57-fold of control levels) and SiO_2_ NP-treated (0.78-fold of control levels) rats (Table 2). Compared to the metabolomic data from recent studies addressing the effects of relevant antibiotics (Doxycycline, Gentamicin, Levofloxacin, Moxifloxacin and Neomycin) [40, 48], a similar or stronger decline in the level of IAA after treatment with most of the antibiotics could be confirmed (Additional file 1: Table S13).

## Discussion

In this work, we provide toxicological, microbial and metabolomics data regarding the impact of orally applied Ag (100 mg/kg body weight/day) and SiO_2_ NP (1000 mg/kg body weight/day) to male Wistar rats. Dosing for both NP was clearly higher than the human daily oral intake of these NP (0.3–0.8 mg/kg body weight/day for SiO_2_ [56] and 0.005 mg/kg body weight /day [57–59] for Ag). Since the individual unintended uptake of NP (e.g. during an accidental scenario or in a working place situation) or the intended uptake as a dietary supplement or medicine can be much higher, testing the limit dose is of relevance for these exposures and could also provide general hazard information. The OECD test guideline no. 407, however, may benefit from updating and adapting for future testing of nanomaterials.

No adverse effects were detectable by a comprehensive assessment of toxicological endpoints, such as loss of body/organ weight or abnormalities in hematology, histology or clinical chemistry (for details see Table 1 and Buesen et al*.* 2014 [51]). However, we found several alterations in the gut microbiome after treatment with Ag or SiO_2_ NP. First, we observed a decrease of the Firmicutes/Bacteroidetes ratio from 2:5 in the controls to 2:1 in Ag-treated rats. Such a decrease has been described as an important parameter for microbial dysbiosis [2, 60]. Consistent with our observations, a nanosilver-induced decrease in the Firmicutes/Bacteroidetes ratio of rodent gut microbiota has been reported before, however, for a lower dosed administration (2.5 mg/kg and up to 36 mg/kg body weight/day) [61, 62]. Other studies showed either no alterations [18, 52], or a significant increase in the Firmicutes/Bacteroidetes ratio as a result of exposure to Ag NP [53]. This diversity in outcomes may result from differences in study designs or handling procedures, such as dosing and type of application, or from specific physico-chemical properties (e.g. primary size/agglomeration, surface modification) of the different nanoparticles used.

Second, we found the phylum Verrucomicrobia and the corresponding genus *Akkermansia* to be virtually absent in Ag-treated rats, whereas *Akkermansia* accounted for 1–4% of the total gut microbiomes in untreated controls and in SiO_2_-treated animals in close accordance to established levels for *Akkermansia municiphila* [54]. Although the observed alterations could not be proven to be statistically significant due to individual variations in the vehicle control group, it should remain under consideration for future studies that *Akkermansia* may be strongly affected by an oral uptake of Ag nanomaterials. Comparable effects have been reported for different metals (Al, Cu, Pb, Cd), where subchronic oral exposures greatly reduced abundance of *Akkermansia* in mice [63] (for review see [64]). *Akkermansia* contributes to the host immune system and stimulates the proliferation of anti-inflammatory regulatory T-cells in mice. In numerous studies, abundance of *A. muciniphila* was found to be reduced in various intestinal diseases, such as Inflammatory Bowel Disease (IBD) or appendicitis as well as in extra-intestinal diseases like obesity, autism or atopy [65–67].

We also found a decrease of *Lactobacillus* after treatment with silver NP. A similar observation has been reported before for Ag-treated rodents [61, 62]. *Lactobacillus* represents a probiotic genus that has been found to protect against IBD [68] and has been described to prevent or treat gastrointestinal disorders in humans [69]. A similar decline in relative abundance of *Lactobacillus* has been found to promote susceptibility to inflammation or mental disorders within different rodent models and in human studies [30, 69–71].

Finally, we detected a significant increase in abundance of the family Prevotellaceae in rats orally treated with Ag NP. This family is known to be composed of four genera, two of which have been identified in the gut*: Prevotella* and *Paraprevotella.* The genus *Paraprevotella* was found to be enriched in the fecal samples of patients with chronic kidney disease [38] and to contribute to autoimmune activation in lupus susceptible mice [72]. The genus *Prevotella* includes more than 40 species, but only three of them have been identified in the gut, with *Prevotella copri* being the generally most abundant one [64]. In mice, *Prevotella* was found to enhance the susceptibility to colitis [73]. In line with this, Chen et al*.* observed an increase of *Prevotella* after oral administration of Ag NP, accompanied by an induction of ulcerative colitis [62]. Vice versa, rats treated with rice straw-derived biochar exhibited reduced abundance of *Paraprevotella* and *Prevotella,* and a decrease of metabolites that can trigger IBD [74]. We therefore assume that the significant increase of *Prevotellacea* we observed in rats after oral treatment with Ag may indicate adverse health effects.

To summarize, after oral treatment with Ag NP, we could identify several alterations of gut microbiota including distinct effects on specific families and genera, which are known to influence the health of the host. We assume that these observed alterations were mediated not only by the nanoparticulate Ag but also by partially dissolved Ag NP. Several studies using acidic solvents and gastric fluids demonstrated that Ag NP undergo dissolution and release silver ions from their surface, depending on pH, particle size and surface coating [75–78]. The results of Axson et al. and Bove et al. indicated that up to 90% of 20 nm silver nanoparticles (NM300K, Bove et al*.*) will be dissolved within minutes through stomach passage [76]. Since the uncoated Ag NP used in our study were determined with an even smaller mean primary particle size of 7 nm (Additional file 1: Table S14), we assume that they were at least partially dissolved after transit through the stomach. However, a high dissolution rate of Ag NP not necessarily leads to a high concentration of free silver ions. Ag NP derived silver ions may bind to the digestive matrices forming Ag-biomolecules or aggregates thereby reducing the available free ions [76, 78], a finding which is supported by a lower bioavailability of silver ions from orally administered Ag NP compared to silver acetate [79].

After oral treatment with SiO_2_ NP, we observed a significant decrease in levels of the family Enterococcaceae and the genus *Enterococcus. Enterococcus* strains have been found to induce significant anti-inflammatory effects and contribute to intestinal epithelium integrity. They were used as probiotics in treatment of Irritable Bowel Syndrome or chronical intestinal diseases and for immune stimulation [80, 81]. A clearly reduced level of *Enterococcus* might therefore be an indicator of an aberrant or unhealthy state.

Additionally, we observed a significant decrease in abundance of the genus *Turicibacter* in SiO_2_-treated animals. In rodents, decreased *Turicibacter* level*s* were found to be consistent with elevated inflammation in the obese status [82]. *Turicibacter* seems to modulate bacterial colonization in the gut. *T. sanguinis* monocolonized in mice was found to regulate numerous genes in the small intestine and colon, which are involved in pathways for steroid and lipid metabolism [83] which suggests an important role in gut microbiota-host co-metabolism. Notably, we also detected significantly decreased levels of the order Peptostreptococcales-Tissierellales and of the genus *Romboutsia* in SiO_2_-treated rats. Species of both genera, *Romboutsia* and *Turicibacter* were found to suppress the growth of pathogens via short chain fatty acid production [84].

On the contrary, we observed an increase in abundance for the families of Oscillospiraceae, Ruminococcaceae, Lachnospiraceae and Prevotellaceae. These results are in line with other reports on oral administration of SiO_2_ NP in mice. Chen et al*.* [62] reported an increase of Lachnospiraceae, Ruminococcaceae, *Oscillobacter* and of the genus *Prevotella*. They also observed an increase in proinflammatory cytokine levels in the colon of mice fed with SiO_2_ NP [62]. Elevated levels of the genus *Prevotella* might be responsible for this increase since *Prevotella* (and its most abundant species in the gut *P. copri*) are known to trigger inflammation [73].

In summary, we have not found an indication for toxicity by classical pathological assessment in the SiO_2_ NP-treated rats [51]. However, the substantial perturbations observed in the gut microbiota *e.g.* the significant increase in Prevotellaceae accompanied by a significant decrease in probiotic genera such as *Enterococcus* and *Turicibacter,* may have a long-term impact on the animals health state.

Metabolome analysis of the rat blood plasma is usually performed to identify metabolite patterns indicative for detrimental effects. Since the gut microbiota affects various metabolic pathways of the host, metabolome results can display unfavorable metabolic profiles and identify potential negative health impacts [85]. In addition to the alterations we found in the gut microbiome, we observed several changes in the plasma metabolome. An overview of the determined effects in comparison to plasma metabolite changes after treatment with different antibiotics is summarized in Table S13 (Additional file 1). A number of 10 different metabolic changes were shown to be significant for Ag NP or SiO_2_ NP treatments of our study, respectively (Table 2), and details about the potentially involved biochemical pathways and specific impacts of the gut microbiome for these metabolites are presented (Additional file 1: Table S15). The observed effects may result from alterations of the gut microbiome or from a direct interaction of the nanomaterial with the host metabolism. After oral treatment with SiO_2_ NP, however, we cannot exclude that a decrease observed in the level of plasma metabolites could also result from adsorption of intestinal metabolites or their precursors to these SiO_2_ NP. The significant increase of allantoin, we observed in rats after Ag NP gavage, may be attributed to a direct interaction of silver with the host organism. Previously, Hadrup et al*.* [86] found a significant increase in allantoin in the urine of female Wistar rats orally exposed to Ag NP or ionic silver (9 mg/kg body weight/day, 28 days), presumably an indication of oxidative stress and concomitant DNA degradation. Interestingly, they could not find metabolic changes in urine of male Wistar rats and suggested that the female rat kidney was more sensitive to Ag NP than the male kidney [86]. In contrast, we observed increased levels of allantoin in the plasma of male rats, a discrepancy that may arise from the different sample source or indicate that the higher dosage of Ag (100 mg/kg body weight/day) compensated for a lower sensitivity of male rats. We further observed a significant increase of pseudouridin levels in SiO_2_ NP-treated rats, which may also result from a stress response upon interaction of SiO_2_ NP with the host [87].

For the pathways of all other metabolites identified with significantly altered levels, a contribution of the gut microbiota has been reported previously (for details see Additional file 1: Table S15). Gut microbiota have been shown to produce a wide range of mammalian neurotransmitter as a mean to communicate with the host [88]. Species of the high abundant genus *Proteus,* which was decreased in abundance in SiO_2_ and Ag-treated rats, have been described to produce norepinephrine [88]. In NP-treated rat plasma, we noted decreased levels of catecholamines, such as 3,4 dihydroxyphenylglycol and epinephrine which are products of norepinephrine. Such kind of effects may be indicative for a disturbed production of neurotransmitters with possible impact on host physiology (Additional file 1: Table S15).

We also found a significant decrease of pantothenic acid (vitamin B_5_) in rat plasma after gavage of SiO_2_. Nearly all species of the phyla Bacteroidota and Proteobacteria are able to produce vitamin B_5_ [89], and we observed a substantial and significant reduction of Proteobacteria in rats exposed to SiO_2_ NP. Recently, changes in the intestinal microbiome were described that led to a reduced B vitamin production in the gut of humans resulting in a lack of pantothenic acid and adverse effects on the immune system as a pro-inflammatory state [90].

Most notably, we found significantly reduced levels of indole-3-acetic acid (IAA) in both, Ag NP- and SiO_2_ NP-treated rats. IAA is a gut-microbiota-derived metabolite produced from dietary tryptophan via the indole pathway [91]. A variety of bacterial species is able to convert tryptophan to indole and indole derivatives including *Lactobacillus* sp﻿ec﻿ie﻿s and *Akkermansia muciniphila* (Additional file 1: Table S15) [91–94]. After administration of antibiotics, a similar decrease of IAA has been found in rodents [40, 48]. However, to our knowledge, a nanoparticle-induced reduction in IAA levels has not been described yet. IAA is a ligand of the aryl hydrocarbon receptor (AhR), an important transcription factor responsible for numerous developmental and tissue-dependent influences on T cell immunity and exerting anti-inflammatory effects in the gut [44]. AhR activation leads to several cellular responses, it orchestrates pathways including hormone and immune response and thus can greatly influence health and disease risks. Different reports suggested a tumor suppressor role for AhR [95]. Lamas et al*.* hypothesized that a NP-induced depletion of AhR ligand producing bacterial strains observed in rodents after long-term treatment with TiO_2_ NP represents the missing link for colon cancer development [2]. Since IAA was also shown to attenuate susceptibility to colitis in mice [44, 93, 96] and to modulate inflammatory responses in hepatocytes and macrophages [97], NP-induced decreased IAA levels may increase the susceptibility of rats to chronic diseases.

Altogether, our results from a limit dose test suggest that oral exposure of rats to either Ag NP or SiO_2_ NP led to changes in the gut bacterial communities that, in turn, can alter the metabolic profile with possible long-term adverse health effects (Table 3). Dose-response studies in the future should be conducted to assess the potential health risks of doses based on the estimated average human dietary intake.

**Table 3:** Summary of key effects on gut microbiome and plasma metabolome determined after orally administration of Ag NP or SiO_2_ NP

Nanomaterial	Effects on the microbiome		Effects on the metabolome		Possible adverse effects
Ag	*Akkermansia*	**↓**			Adverse effects on the immune system [67]
Ag			Allantoin	↑	Sign of oxidative stress [86]
SiO_2_	*Enterococcus*	**↓**			Adverse effects on the immune system [81]
SiO_2_	*Turicibacter*	**↓**			Elevated inflammation [82]
SiO_2_	*Romboutsia*	**↓**			Increased growth of pathogens [84]
SiO_2_	Proteobacteria	**↓**	Panthotenic acid (Vitamine B5)	**↓**	Adverse effects on the immune system [90]
SiO_2_			Pseudouridine	↑	Sign of stress response [87]
Ag/SiO_2_	Prevotellacea	↑			Members of this family can trigger IBD [73]
Ag/SiO_2_	*Proteus*	**↓**	Catecholamines	**↓**	Disturbed production of neurotransmitters [88]
Ag/SiO_2_	*Lactobacillus*	**↓**	Indole-3-acetic-acid	**↓**	Enhanced susceptibility to chronic intestinal diseases [93, 96]

Ag: effects only observed in rats treated with Ag NP, SiO_2_: effects only observed in rats treated with SiO_2_ NP, Ag/SiO_2_: similar effects observed in rats treated either with Ag NP or with SiO_2_ NP

## Conclusions

In this study, the effects of an oral exposure of two well-characterized nanoparticles, SiO_2_ NP and Ag NP, were investigated in male Wistar rats. We did not find any treatment-related signs of toxicity in histopathology and clinical pathology, but we observed significant changes in gut microbiota, which have been related to adverse health effects, and changes in plasma metabolites, which are associated with microbiome changes. Our results demonstrate that an oral uptake of SiO_2_ NP or Ag NP can affect gut microbiota in vivo. The resulting changes of the intestinal microbiome were specific for each type of NP. Some of the bacterial families and genera, which are known to play important roles in keeping individuals healthy, were found to be reduced in the NP-treated animals. Table 4 summarizes the most important findings from this study (Table 4).

**Table 4:** Key findings of the combined microbiome-/metabolome profiling for toxicological endpoints

Endpoint	Key finding
Clinical pathology	High dose oral application of SiO_2_ and Ag nanoparticles had no detectable effects on established clinical pathology endpoints in male Wistar rats
Gut microbiome	Ingested SiO_2_ and Ag nanoparticles altered the gut microbiome significantly
	SiO_2_ and Ag NP influence the level of microbial genera some of which are known to mediate probiotic or adverse effects
	The gut microbiome is a sensitive indicator for possible hazards caused by orally administrated NP
Plasma metabolome	Orally applied SiO_2_ and Ag NP led to changes in the level of several plasma metabolites known to be crucial for human health
	Key plasma metabolites (e.g. gut-microbiota derived IAA) are suitable markers for potential adverse effects induced by orally applied NP
Combined gut microbiome and plasma metabolome	The combination of gut microbiome and plasma metabolome profiling has a strong potential as a sensitive tool to disclose early detrimental effects of ingested NP

We found significant and important changes in the plasma metabolomes of NP-treated rats, which were specific for each nanoparticle. Importantly, a significant decrease in levels of the AhR ligand IAA was found after the oral gavage of both of the NP. This is the first report confirming a NP-induced reduction of AhR ligands in vivo, an observation that should be carefully attended for health and disease risk assessments.

Albeit no signs of toxicity were found in the rats after 28 days of NP exposure, our results suggest that long-term effects are conceivable and should be taken into consideration. Future studies with a combined analysis of gut microbiota and plasma metabolome over protracted periods of nanomaterial exposure and dose ranges, reflecting more the average dietary intake of humans will help to understand the relevance of the altered metabolic profiles.

## Methods

### Test substances and particle characterization

The test substances were selected from the set of the nanoGEM study [98, 99] *i.e.* SiO2 NP (SiO_2_.naked NP without different surface modifications; Levasil® 200) and Ag NP (Ag50 EO NP). SiO_2_ was supplied by BASF SE, Ludwigshafen, Germany, Ag NP by Bayer Material Sciences, Leverkusen, Germany. The test substances were delivered as dispersions and were characterized in detail in accordance with the physico-chemical endpoints described in the guidance on information requirements for nanomaterials [100] to EU regulation No. 1907/2006 on the Registration, Evaluation, Authorization and Restriction of Chemicals [101]. The following test substance properties were determined making use of the indicated methodologies [98, 102, 103].

Mean primary particle size and primary particle size (PPS) distribution (TEM); hydrodynamic particle size in water [dynamic light scattering (DLS) and analytical ultracentrifugation (AUC)]; particle morphology [light microscopy and scanning electron microscopy (SEM)]; crystallinity [X-ray diffraction (XRD)]; surface chemistry, purity, and crystalline phase [X-ray photoelectron spectroscopy (XPS)]; organic surface functionalization [secondary ion mass spectrometry (SIMS)]; iso-electric point and zeta-potential (electrophoretic mobility titration); surface reactivity and radical formation potential (Electron spin resonance (ESR) making use of centrophenoxine (CPH) or dimethyl-pyrroline-N-oxide (DMPO) spin traps). For the test material SiO_2_ and Ag, the state of agglomeration in the test substance vehicle of the 28-day oral toxicity studies, *i.e.*, phosphate buffered saline (PBS) supplemented with 1 g/L bovine serum albumin (BSA; in the following: ‘PBS + BSA’), was determined by laser diffraction and AUC.

An overview of the primary and secondary physico-chemical properties of both test substances is provided in Table S14 (Additional file 1), which has been adapted from [102, 103]. Further information on the preparation and characterization of the set of nanoGEM test substances is available from [98].

### Preparation of test substances

The original test suspensions, as provided by the suppliers, were shaken and mixed for 2 min using a vortex mixer to ensure a homogeneous distribution of particles. Next, the desired amount of test substance was weighed and then filled up with the test substance vehicle PBS + BSA to obtain uniform test substance solutions of 10 %wt solutions. Test substance preparations were produced daily and were kept homogenous until administration by continuous stirring with a magnetic stirrer.

Since nanoparticles can agglomerate and sediment quickly in suspensions and this can considerably affect the final effective dosage reaching the target organism, it is essential to assess the homogeneity of test substances and to verify the effective concentration in the test substance preparations. Therefore, at the onset of the administration period, homogeneity and concentration control analyses of all test substance suspensions (‘as delivered’ and ‘as prepared’ in PBS + BSA) were performed by inductively coupled plasma-optical emission spectrometry (ICP–OES). For this purpose, three separate samples of the test substance preparations were taken from the bottom, middle, and top layers of the vials (which would necessarily have the same test substance concentrations in homogenous suspensions). The content of the metallic element of the respective test substances (*i.e.* silicon in the case of the SiO_2_) was measured and the mass of the entire test substance molecule derived from these measurements. The mass of the substances used for surface functionalization was considered to be negligible [104].

### Test substance characterization

Both test substances were well dispersed in water and had average agglomeration numbers (AAN, *i.e.*, the average number of primary particles in the agglomerate) of 1. SiO_2_ remained stable in PBS + BSA with only minimal agglomeration, but also here BSA adsorption was recorded. When diluted in DMEM + FCS, SiO_2_ was moderately agglomerated (AAN = 28). The iso-electric point of SiO_2_ was at a pH value below 1.

When diluted in DMEM + FCS, Ag remained well dispersed (AAN = 1); the particle size in PBS + BSA was not determined. The iso-electric point of Ag was at a pH value of 2.5.

### Performance, statistical analysis and interpretation of findings of the animal study

The 28-day oral toxicity studies were performed with male Wistar rats (Crl:WI(Han), Charles River Laboratories, Sulzfeld, Germany). The animal facility, in which all animal work was performed, holds a certificate from the International Association for Assessment and Accreditation of Laboratory Animal Care (AAALAC). The animal studies were performed with approval of the local authorizing agency for animal experiments (Landesuntersuchungsamt Rheinland-Pfalz, Koblenz, Germany) on 30th of January 2009, as referenced by the approval number 23 177-07/G 08-3-007, and study protocols complied with the respective guidelines. For SiO_2_, the experiment was performed as limit test, applying a single dose level of 1000 mg/kg body weight/day (cf. paragraph 18 of OECD TG 407) [49]. Ag was applied at a dose level of 100 mg/kg body weight/day. The test substance preparations were administered daily by oral gavage over a period of four weeks to groups of five male rats. Control groups of 5 male animals received only the vehicle PBS + BSA. Animals were regularly subjected to detailed clinical observations, assessment of food and water consumption and the body weight; hematological and clinical chemical examinations were performed toward the end of the administration period. Upon completion of the administration period, all animals were subjected to a full, detailed gross necropsy and histopathological examinations were performed on all organs listed in OECD test guideline no. 407, paragraph 43 [49].

### Metabolome analysis with MetaMap® Tox methodology

As described by van Ravenzwaay et al*.* and Kamp et al*.* [45, 46], EDTA-K3 blood samples of all rats taken on day of sacrifice were analyzed in regard to their metabolite profiles upon metabolite extraction by a proprietary method: GC–MS and LC–MS/MS were applied for broad profiling and hormone measurement. The method resulted in 225 semi-quantitative analytes, 171 of which were chemically identified and 54 were structurally unknown. Analysis of the recorded metabolite profiles was performed making use of the MetaMap® Tox database [105], (cf. Information box MetaMap® Tox methodology).

The data were analyzed by univariate and multivariate statistical methods. The day-stratified heteroscedastic *t*-test ("Welch test") was applied to compare metabolite levels of NP-treated groups with respective controls. For all metabolites, changes were calculated as the ratio of the mean of metabolite levels in individual rats in a treatment group relative to mean of metabolite levels in rats in a matched control group. The *p* values, *t*-values, and ratios of corresponding group medians were collected as metabolic profiles and fed into the MetaMap®Tox database [106]. In the database, all treatment groups were compared to the controls of the corresponding study. The profile strength of the metabolic profile was addressed. This parameter represents the rounded down average of absolute medians of *t*-values and does not only include the absolute number of significantly changed metabolites, but also the magnitude of the respective changes. The best balance for finding the maximum number of truly regulated metabolites, while minimizing the number of false positive regulated metabolites, was obtained at a *p* value of 0.15. Therefore, for pattern recognition *p* values up to 0.2 were used in the database for plasma. For a detailed description on the use of statistics in MetaMap®Tox see van Ravenzwaay et al*.* [106].

### Feces sampling for microbiome analyses

For this purpose, the animals were transferred into metabolism cages (no food or drinking water provided) in the afternoon preceding the day fixed sampling. On the following morning, urine samples were taken for the scheduled urine analysis, whereas feces were directly frozen. All feces samples were stored at − 80 °C for further analyses.

### Isolation of bacterial DNA from feces

Feces of male rats were collected towards the end of the administration period, *i.e.* on day 25 of the study and stored at − 80 °C. DNA isolation from rat feces was performed using the innuSPEED Stool DNA Kit (analytikjena, Jena, Germany) according to the instructions of the manufacturer. In particular, we used samples of 300 mg feces, added 1 mL of lysis solution (provided in the innuSPEED stool kit DNA kit) and homogenized it for about 30 min. After sample cleanup, binding and washing according to protocol 2 of the innuSPEED stool DNA Kit manual, DNA was eluted from the column using 2 × 100 µl of elution buffer, and subsequently stored at − 20 °C. Quality and purity of the isolated genomic DNA was confirmed by agarose gel-electrophoresis and spectrophotometry on the NanoDrop 2000 device (Fisher Scientific, Schwerte, Germany). DNA concentration was estimated with the Qubit 2.0 instrument applying the Qubit dsDNA HS Assay (Life Technologies, Invitrogen division, Darmstadt, Germany).

### Next generation sequencing for mircobiome community analysis

For NGS library preparation, the recommended protocol for preparing 16S ribosomal RNA gene amplicons for the Illumina MiSeq system was used (Illumina Inc., San Diego, CA, USA). The suggested universal bacterial primers (Bakt_341F:5′-CCTACGGGNGGCWGCAG-3′ and Bakt_805r:5′-GACTACHVGGGTATCTAATCC-3′) were utilized for amplifying the V3 and V4 hypervariable regions of the bacterial 16S rRNA gene with polymerase chain reaction (PCR) using the KAPA Hifi HotStart Ready Mix (Roche Diagnostics Deutschland, Mannheim, Germany). Purity and exact fragment size of amplicons were determined with the Caliper GX system using the HT DNA High Sensitivity LabChip Kit (PerkinElmer, Rodgau, Germany). In a second PCR, sample-specific “barcode”-primers and adapter sequences were attached. All libraries were normalized and pooled for an Illumina MiSeq sequencing run using the MiSeq Reagent Kit version (v.) 3 (Illumina, San Diego, CA, USA) with marginally overlapping 300 base pairs (bp) paired end reads.

### Bioinformatics and statistical analysis

The quality of the 16S rRNA gene sequencing data was analyzed using FastQC v0.11.5 [107]. The reads were quality trimmed using Sickle v1.33 (https://github.com/najoshi/sickle) and analyzed with Qiime2 (2020.11) [108]. Briefly, the reads were imported into the Qiime2 pipeline and demultiplexed. The included DADA2 pipeline was then used to denoise the 18,305,252 paired sequence reads, remove chimeric sequences, and infer the amplicon sequence variants (ASV) [109]. Taxonomy classification of ASVs was then performed against the full-length Silva database (v138, 99% identity cutoff) using the q2-feature-classifier [110, 111]. The PCoA of Bray–Curtis distance was calculated using the ASV data. Qiime2 view (https://view.qiime2.org/) was used for visualization. Relative abundance of ASVs from level 2 (phylum) to 6 (genus) were used for further analysis. The differences in the relative abundance between the three groups were analyzed at each level with the Mann–Whitney U test (alpha = 0.05). Alpha diversity (i.e. richness and evenness) was calculated ASV-based with the Shannon–Wiener and the Inverse Simpson Index for biodiversity using Microsoft Excel®, and evaluated by Student’s *t-*test with significance defined as *p* < 0.05. Rarefaction curves generated from the ASVs suggested adequate sampling coverage was achieved in all samples (Additional file 1: Fig. S5). Beta-diversity was calculated with Bray–Curtis distance values, and Principal Coordinates Analysis (PCoA) was visualized using R v4.0.2 [112].

## Supplementary Information

**Additional file 1**. Changes in the composition of intestinal microbes in male Wistar rats after a 25-day gavage with the vehicle: The differences in the microbiota composition of untreated rats at day 0 compared to vehicle treated controls at day 25 were further analyzed at the taxonomic level of phyla and classes. **Table S1:** Body weight. **Table S2:** Hematology: Red blood cell and coagulation parameters. **Table S3:** Hematology: White blood cell parameters. **Table S4:** Clinical chemistry in blood samples. **Table S5:** Relative abundance of bacterial phyla (≥ 0.1% in at least one group) in the feces of male Wistar rats. **Table S6:** Relative abundance of bacterial classes (≥ 0.1% in at least one group) in the feces of male Wistar rats. **Table S7:** Relative abundance of bacterial phyla (≥ 0.1% in at least one group) in the feces of male Wistar rats. **Table S8:** Relative abundance of bacterial classes (≥ 0.1%) in the feces of male Wistar rats. **Table S9:** Relative abundance of bacterial order (≥ 0.1% in at least one group) in the feces of male Wistar rats. **Table S10:** Relative abundance of bacterial family (≥ 0.1 % in at least one group) in the feces of male Wistar rats. **Table S11:** Relative abundance of bacterial genera (≥ 1% in at least one group) in the gut microbiota of male Wistar rats. **Table S12:** Individual sample and median values for the relative abundance of selected most abundant bacterial genera (≥ 1% in at least one group) in the gut microbiota of male Wistar rats. **Table S13:** Overall comparison of plasma metabolite changes in male Wistar rats after 28 days of treatment with Ag NP or SiO2 NP and changes observed after treatments with different antibiotics. **Table S14:** Physicochemical characterization of the test substances (adapted from: Hellack et al., 2012; Wohlleben et al., 2013 [8, 9]). **Table S15:** Altered plasma metabolite pathways in Ag NP or SiO2 NP-treated rats and their functional relationship with the gut microbiota. **Fig. S1:** Alpha diversity of the gut microbiota in male Wistar rats before (untreated) and after exposure to vehicle, Ag NP (Ag50) or SiO2 NP (SiO2), shown as Shannon-Wiener index (a) or Inverse Simpson index (b). **Fig. S2:** Beta-diversity of gut microbiota visualized as PCoA plots. Beta-diversity illustrated for samples of untreated male Wistar rats (at day 0) compared to the same animals after 25 days of gavage treatment with vehicle, Ag NP or SiO2 NP (a), or samples from male Wistar rats after 25 days of gavage treatment only to compare treatments with vehicle, Ag NP and SiO2 NP (b). **Fig. S3:** Relative abundance of bacterial phyla (a) and classes (b) in the gut microbiota (≥ 1%) of male Wistar rats before (untreated) and after a 25-day gavage of PBS + BSA (vehicle). **Fig. S4:** Scatter plots obtained for selected most abundant genera after exposure to either Ag or SiO2 NP (median relative abundance was ≥ 1% in at least one group). **Fig. S5:** Rarefaction curves of all animals used for the analyses (samples of untreated rats at day 0; vehicle controls, Ag NP and SiO2NP-treated samples at day 25, respectively).

## Data Availability

All datasets used and/or analysed during the current study are available from the corresponding author on reasonable request.
