# Supplementary material for: Gut microbiome and plasma metabolome changes in rats after oral gavage of nanoparticles: sensitive indicators of possible adverse health effects
Source: Part Fibre Toxicol. 2022 Mar 23;19:21. doi: 10.1186/s12989-022-00459-w (PMC8941749; doi:10.1186/s12989-022-00459-w)
Supplement: Supplementary file 1 — Additional file 1. Changes in the composition of intestinal microbes in male Wistar rats after a 25-day gavage with the vehicle: The differences in the microbiota composition of untreated rats at day 0 compared to vehicle treated controls at day 25 were further analyzed at the taxonomic level of phyla and classes. Table S1: Body weight. Table S2: Hematology: Red blood cell and coagulation parameters. Table S3: Hematology: White blood cell parameters. Table S4: Clinical chemistry in blood samples. Table S5: Relative abundance of bacterial phyla (≥ 0.1% in at least one group) in the feces of male Wistar rats. Table S6: Relative abundance of bacterial classes (≥ 0.1% in at least one group) in the feces of male Wistar rats. Table S7: Relative abundance of bacterial phyla (≥ 0.1% in at least one group) in the feces of male Wistar rats. Table S8: Relative abundance of bacterial classes (≥ 0.1%) in the feces of male Wistar rats. Table S9: Relative abundance of bacterial order (≥ 0.1% in at least one group) in the feces of male Wistar rats. Table S10: Relative abundance of bacterial family (≥ 0.1 % in at least one group) in the feces of male Wistar rats. Table S11: Relative abundance of bacterial genera (≥ 1% in at least one group) in the gut microbiota of male Wistar rats. Table S12: Individual sample and median values for the relative abundance of selected most abundant bacterial genera (≥ 1% in at least one group) in the gut microbiota of male Wistar rats. Table S13: Overall comparison of plasma metabolite changes in male Wistar rats after 28 days of treatment with Ag NP or SiO2 NP and changes observed after treatments with different antibiotics. Table S14: Physicochemical characterization of the test substances (adapted from: Hellack et al., 2012; Wohlleben et al., 2013 [8, 9]). Table S15: Altered plasma metabolite pathways in Ag NP or SiO2 NP-treated rats and their functional relationship with the gut microbiota. Fig. S1: Alpha diversity of the gut microbiota in male W [file 12989_2022_459_MOESM1_ESM.docx]

**Gut microbiome and plasma metabolome changes in rats after oral gavage of nanoparticles - sensitive indicators of possible adverse health effects**

**Robert Landsiedel^1#^, Daniela Hahn^2#^, Rainer Ossig^2^, Sabrina Ritz^2^, Lydia Sauer^2^, Roland Buesen^1^, Sascha Rehm^3,4^, Wendel Wohlleben^5^, Sibylle Groeters^1^, Volker Strauss^1^, Saskia Sperber^1^, Haleluya Wami^6^, Ulrich Dobrindt^6^, Karola Prior^7^, Dag Harmsen^7^, Bennard van Ravenzwaay^1#^, Juergen Schnekenburger^2#^**

^#^ Equally contributing first and senior authors, respectively

^1^ Experimental Toxicology and Ecology, BASF SE, 67056 Ludwigshafen am Rhein, Germany

^2^ Biomedical Technology Center of the Medical Faculty, University of Muenster, 48149 Muenster, Germany

^3^ HB Technologies AG, 72076 Tuebingen, Germany

^4^ Medical Data Integration Center, University Tuebingen, 72072 Tuebingen, Germany

^5^ Polymer Physics, BASF SE, 67056 Ludwigshafen am Rhein, Germany

^6^ Institute of Hygiene, University of Muenster, 48149 Muenster, Germany

^7^ Department of Periodontology and Operative Dentistry, University Hospital Muenster, 48149 Muenster, Germany

Corresponding author: Dr. Juergen Schnekenburger,

Biomedical Technology Center of the Medical Faculty

University of Muenster

Mendelstrasse 17

48149 Muenster, Germany

Telephone: +49(0)2518352534

Email: [schnekenburger@uni-muenster.de](mailto:schnekenburger@uni-muenster.de)

**Supplementary Information**

**Changes in the composition of intestinal microbes in male Wistar rats after a 25-day gavage with the vehicle**

The differences in the microbiota composition of untreated rats at day 0 compared to vehicle treated controls at day 25 were further analyzed at the taxonomic level of phyla and classes (Fig. S3). In the untreated control (UC), Bacteroidota and Firmicutes were detected as the two predominant phyla, which account for nearly 91% of the total microbiome with an average ratio of approx. 1:1 Firmicutes:Bacteroidota. The third largest phylum identified was the Verrucomicrobia contributing a fraction of 6.2% to the total quantity, followed by Proteobacteria (1.9%). All other identified phyla were below 1% among which Actinobacteriota (0.2%) and Patescibacteria (0.2%) were the most prominent (Fig. S3a, Table S5).

After the 25-day gavage of the vehicle PBS + BSA (VC), we observed distinct alterations in the gut microbial communities (Fig. S3). The level of Bacteroidota decreased from 47.7 % to 23.5% accompanied by an increase of Firmicutes from 43.1% to 57.5%, which led to a shift in the ratio of Firmicutes:Bacteroidetes towards 2.5:1. Most strikingly, the level of Proteobacteria increased from 1.9% towards 16.8% in the vehicle-treated animals. In contrast, the relative abundance of Verrucomicrobiota decreased from 6.2% towards 1.3% (Fig. S3a, for further details see Table S5).

At the level of classes, the described changes were dominated by a decrease of Bacteroidia from 47.7% in UC towards 23.5% in VC and an increase of the class Bacilli from 6.4% to 22.6%. Moreover, a fundamental enrichment of the class Gammaproteobacteria (from 0.2% in UC to 16.7% in VC) seems to be responsible for the observed change of phylum Proteobacteria. (Fig. S3b, for further details see Table S6).

As for the vehicle control, we observed a similar differentiation of microbiota also between each of the NP-treated groups and the UC group (Fig. S2a). Since for all rats of each study group, the cage conditions and daily intake of food and water were similar, the sexual maturation during the 25-day gavage or the treatment with vehicle can be the possible cause for the observed differentiation of the microbial profiles from UC. Recently, Han *et al.* studied the impact of 4-epi-oxytetracycline in normal saline on the gut microbiota in a 28-day oral study using Wistar rats. Comparable to the data presented here, they found a Firmicutes:Bacteroidota ratio of 2.2:1 (55% Firmicutes, 25% Bacteroidota) in control male rats after 28 days. Different from our study, they collected the feces once every four days. They also reported progressive changes in the gut microbiota from day 0 to day 28 in their control group.^[1]^ With regard to these results, the differences we found between the untreated controls (collected at day 0 of the study) and the vehicle-treated controls (collected at day 25 of the study) were most probably determined by animal aging and development rather than by effects of the treatment. At the same time, also the inter-animal variations within treatment groups were enhanced during this 25-day aging period (Fig. S2a). Similar aging effects, *i.e*. an increase in the Firmicutes:Bacteroidetes ratio from birth to adulthood has been previously reported also for the human gut microbiota.^[2]^ In order to avoid an inclusion of these possible aging effects in our analysis, only those data obtained for the vehicle-treated animals after day 25 were used as relevant controls for the nanoparticle-treated animals throughout the study.

**Supplementary Tables**

**Table S1:** **Body weight**

|  | **Male animals** | | |
| --- | --- | --- | --- |
| **Day** | **VC** | **SiO_2_** | **Ag** |
| 0 | 164 | 167 | 169 |
| 7 | 204 | 211 | 207 |
| 14 | 239 | 248 | 249 |
| 21 | 265 | 275 | 277 |
| 28 | 286 | 295 | 296 |

SiO_2_, Ag nanoparticles and vehicle control (VC) were applied daily for 28 days; body weight (g), expressed as mean of test group (N = 5), data shown for SiO_2_ NP had already been published by Buesen *et al.* 2014 (denominated “SiO_2_ naked” in that publication).^[3]^

**Table S2: Hematology: Red blood cell and coagulation parameters**

|  | **VC** | **SiO_2_** | **Ag** | **Historical control range ^a^** |
| --- | --- | --- | --- | --- |
| **RBC; tera/L** | 7.94 | 8.27 | 8.40* | 7.59-8.60 |
| **HGB; mmol/L** | 8.9 | 8.8 | 9.0 | 8.6-9.5 |
| **HCT; L/L** | 0.432 | 0.425 | 0.440 | 0.384-0.432 |
| **MCV; fL** | 54.5 | 51.4 | 52.4 | 48.1-53.3 |
| **MCH; fmol** | 1.12 | 1.07 | 1.07 | 1.06-1.19 |
| **MCHC; mmol/L** | 20.58 | 20.81 | 20.41* | 20.43-23.73 |
| **RET; %** | 2.2 | 2.1 | 1.7* | 1.4-3.1 |
| **PLT; giga/L** | 836 | 792 | 911 | 791-1,025 |
| **HQT; sec** | 37.1 | 33.9 | 34.9 | 33.3-39.6 |

Data were determined on day 29 and expressed as mean of test group (N = 5), data shown for SiO_2_ nanoparticles had already been published by Buesen *et al.* 2014. (denominated “SiO_2_ naked” in that publication)^[3]^ *RBC* red blood cells, *HGB* hemoglobin, *HCT* hematocrit, *MCV* mean corpuscular volume, *MCH* mean corpuscular hemoglobin, *MCHC* mean corpuscular hemoglobin concentration, *RET* reticulocytes, *PLT* platelets, *HQT* prothrombin time (Heptatoquick® test), *Units* giga/L = 10^9^/liter; tera/L = 10^12^/liter; fL = fentoliter; mmol/L = millimole/liter; fmol = femtomole; L/L = liter/liter; sec = seconds Significance, as compared to the control group: ** p* ≤ 0.5; *** p* ≤ 0.1 a Historical control range (*N* = 37), unpublished in-house data

**Table S3: Hematology: White blood cell parameters**

|  | **VC** | **SiO_2_** | **Ag** | **Historical control range ^a^** |
| --- | --- | --- | --- | --- |
| **WBC; giga/L** | 6.30 | 5.86 | 5.86 | 4.38-7.90 |
| **LUC; giga/L** | 0.04 | 0.05 | 0.03 | 0.01-0.05 |
| **LUC; %** | 0.6 | 1.0 | 0.5 | 0.3-0.8 |
| **Neut.; %** | 12.8 | 16.6 | 15.5 | 9.4-16.6 |
| **Lymph.; %** | 83.0 | 78.7 | 79.6 | 79.1-87.0 |
| **Mono.; %** | 1.7 | 2.4 | 2.3 | 0.9-2.5 |
| **Eos.; %** | 1.5 | 1.0* | 1.7 | 1.1-2.8 |
| **Baso.; %** | 0.4 | 0.3 | 0.4 | 0.0-0.9 |

Data were determined on day 29 and expressed as mean of test group (N=5), data shown for SiO_2_ had already been published by Buesen *et al.* 2014 (denominated “SiO_2_ naked” in that publication).^[3]^ *WBC* white blood cells, *LUC* large unstained cells, *Neut.* polymorphonuclear neutrophils, *Lymph.* lymphocytes, *Mono.* monocytes, *Eos.* eosinophils, *Baso.* Basophils, *Units*: giga/L = 10^9^/liter, Significance, as compared to the control group: * *p* ≤ 0.5, a Historical control range (*N* = 37), unpublished in-house data

**Table S4: Clinical chemistry in blood samples**

|  | **VC** | **SiO_2_** | **Ag** | **Historical control range ^a^** |
| --- | --- | --- | --- | --- |
| **ALT; µkat/L** | 0.80 | 0.76 | 0.77 | 0.53-0.89 |
| **AST; µkat/L** | 1.99 | 2.10 | 1.86 | 1.46-2.42 |
| **AP; µkat/L** | 2.35 | 1.99 | 2.68 | 1.50-2.80 |
| **gGT; nkat/L** | 0 | 0 | 0 | 0-13 |
| **TP; g/L** | 60.20 | 62.34 | 61.02 | 59.09-65.07 |
| **Alb; g/L** | 37.53 | 38.20 | 38.00 | 35.28-38.41 |
| **Glob; g/L** | 22.67 | 24.13 | 23.02 | 22.15-28.70 |
| **Na; mmol/L** | 141.8 | 140.5 | 141.4 | 139.1-146.0 |
| **K; mmol/L** | 4.45 | 4.52 | 4.58 | 4.29-4.91 |
| **Cl; mmol/L** | 101.7 | 100.4 | 101.6 | 99.2-104.0 |
| **INP; mmol/L** | 2.15 | 2.01 | 2.24 | 1.88-2.39 |
| **Ca; mmol/L** | 2.53 | 2.53 | 2.57 | 2.42-2.70 |
| **Urea; mmol/L** | 5.77 | 6.23 | 6.36 | 4.69-7.67 |
| **Crea; µmol/L** | 45.1 | 47.5 | 46.3 | 43.9-52.5 |
| **TBIL; µmol/L** | 1.40 | 1.38 | 1.29 | 1.46-2.63 |
| **Hapt.; ng/mL** | 234.02 | 413.56* | 287.83 | 265.2-1,074.0 |
| **α2m; ng/mL** | 11.08 | 14.29 | 12.72 | 8.92-33.77 |

Enzymes, substrates, electrolytes, acute phase proteins were determined on day 29, expressed as mean of test group (N=5), data shown for SiO_2_ nanoparticles had already been published by Buesen *et al.* 2014 (denominated “SiO_2_ naked” in that publication).^[3]^ a: unpublished in-house data, Significance, as compared to the control group: p ≤ 0.5 is indicated with *, ALT = alanine aminotransferase; AST = aspartate aminotransferase; AP = alkaline phosphatase; gGT = gamma glutamyl transferase; TP = total protein; Alb. = albumin; Glob. = globulins; Na = sodium; K = potassium; Cl = chloride; Hapt. = haptoglobin; α2m = α2-macroglobin. *Units*: µkat/L = microkatal per liter; nkat/L = nanokatal per liter; ng/mL = nanogram per milliliter; g/L = gram per liter; mmol/L = millimole per liter

**Table S5: Relative abundance of bacterial phyla (≥ 0.1% in at least one group) in the feces of male Wistar rats**

UC: untreated control animals at day 0, VC: 25 days vehicle-treated animals.

Mean values expressed as % of the total gut microbiota. Values are shown for phyla with relative abundance ≥ 0.1% in at least one group.

**Table S6: Relative abundance of bacterial classes (≥ 0.1% in at least one group) in the feces of male Wistar rats**

UC: untreated control animals at day 0, VC: 25 days vehicle-treated animals.

Mean values expressed as % of the total gut microbiota. Values are shown for classes with relative abundance ≥ 0.1% in at least one group.

**Table S7: Relative abundance of bacterial phyla (**≥ **0.1% in at least one group) in the feces of** **male Wistar rats**

**VC**

**mean**

**mean**

**p-value**

**mean**

**p-value**

Actinobacteriota

0.34%

0.42%

0.232

0.16%

0.059

Bacteroidota

23.46%

30.35%

0.087

25.23%

0.377

Firmicutes

57.45%

60.80%

0.174

69.56%

0.008 *

Patescibacteria

0.16%

0.12%

0.232

0.01%

0.005 *

Proteobacteria

16.83%

7.82%

0.125

1.32%

0.038 *

Verrucomicrobiota

1.26%

0.04%

0.174

2.38%

0.087

**Phylum**

**Ag**

**SiO_2_**

VC: vehicle-treated controls, Ag: Ag NP-treated animals, SiO_2_: SiO_2_ NP-treated animals. Mean values expressed as % of the total gut microbiota, N = 5 for each treatment group, asterisks mark significant changes, *p*-values were obtained by using the Mann-Whitney-U-test.

**Table S8: Relative abundance of bacterial classes (**≥ **0.1%) in the feces of male Wistar rats**

**VC**

**mean**

**mean**

**p-value**

**mean**

**p-value**

Actinobacteriota: Actinobacteria

0.09%

0.11%

0.301

0.06%

0.125

Actinobacteriota: Coriobacteriia

0.25%

0.32%

0.302

0.10%

0.024 *

Bacteroidota: Bacteroidia

23.46%

30.35%

0.087

25.23%

0.377

Firmicutes: Bacilli

22.58%

18.02%

0.174

17.02%

0.174

Firmicutes: Clostridia

34.87%

42.77%

0.125

52.54%

0.038 *

Patescibacteria: Saccharimonadia

0.16%

0.12%

0.232

0.01%

0.005 *

Proteobacteria: Alphaproteobacteria

0.09%

0.16%

0.377

0.10%

0.377

Proteobacteria: Gammaproteobacteria

16.74%

7.66%

0.125

1.23%

0.059

Verrucomicrobiota: Verrucomicrobiae

1.26%

0.04%

0.174

2.38%

0.087

**Class**

**Ag**

**SiO_2_**

VC: vehicle-treated controls, Ag: Ag NP-treated animals, SiO_2_: SiO_2_ NP-treated animals. Mean values expressed as % of the total gut microbiota, N = 5 for each treatment group, asterisks mark significant changes, *p*-values were obtained by using the Mann-Whitney-U-test*.*

**Table S9: Relative abundance of bacterial order (**≥ **0.1% in at least one group) in the feces of male Wistar rats**

**VC**

**mean**

**mean**

**p-value**

**mean**

**p-value**

Coriobacteriia: Coriobacteriales

0.25%

0.32%

0.301

0.10%

0.024*

Bacteroidia: Bacteroidales

23.03%

29.90%

0.059

25.06%

0.301

Bacilli: Bacillales

0.21%

0.56%

0.377

0.27%

0.458

Bacilli: Erysipelotrichales

1.45%

2.95%

0.008*

0.69%

0.024*

Bacilli: Lactobacillales

19.48%

12.77%

0.087

15.31%

0.232

Bacilli: RF39

0.31%

1.05%

0.301

0.68%

0.087

Bacilli: Staphylococcales

1.12%

0.65%

0.458

0.02%

0.014*

Clostridia: Christensenellales

1.00%

0.25%

0.087

1.50%

0.301

Clostridia: Clostridia_UCG-014

6.72%

10.71%

0.087

3.94%

0.059

Clostridia: Clostridia_vadinBB60

_group

0.31%

0.54%

0.059

0.47%

0.232

Clostridia: Clostridiales

0.51%

0.85%

0.174

0.08%

0.232

Clostridia: Lachnospirales

10.07%

8.80%

0.301

18.53%

0.038*

Clostridia: Monoglobales

0.13%

0.08%

0.301

0.20%

0.174

Clostridia: Oscillospirales

13.20%

18.88%

0.005*

26.64%

0.038*

Clostridia: Peptococcales

0.02%

0.03%

0.125

0.16%

0.005*

Clostridia: Peptostreptococcales

-Tissierellales

2.75%

2.36%

0.232

0.89%

0.005*

Saccharimonadia: Saccharimonadales

0.16%

0.12%

0.232

0.01%

0.005*

Alphaproteobacteria: Rhodospirillales

0.08%

0.16%

0.301

0.08%

0.301

Gammaproteobacteria: Enterobacterales

16.71%

7.62%

0.125

1.17%

0.059

Verrucomicrobiae: Verrucomicrobiales

1.26%

0.04%

0.174

2.38%

0.087

**Order**

**Ag**

**SiO_2_**

VC: vehicle-treated controls, Ag: Ag NP-treated animals, SiO_2_: SiO_2_ NP-treated animals.
Mean values expressed as % of the total gut microbiota, N = 5 for each treatment group, asterisks mark significant changes, *p*-values were obtained by using the Mann-Whitney-U-test.

**Table S10: Relative abundance of bacterial family (**≥ **0.1 % in at least one group) in the feces of male Wistar rats**

**VC**

**mean**

**mean**

**p-value**

**mean**

**p-value**

Coriobacteriales: Eggerthellaceae

0.23%

0.24%

0.458

0.09%

0.024*

Bacteroidales: Bacteroidaceae

4.60%

6.85%

0.087

6.58%

0.059

Bacteroidales: Muribaculaceae

14.63%

15.63%

0.301

12.45%

0.174

Bacteroidales: Prevotellaceae

1.94%

5.62%

0.024*

4.52%

0.014*

Bacteroidales: Tannerellaceae

1.87%

1.80%

0.301

1.50%

0.232

Bacillales: Bacillaceae

0.13%

0.13%

0.623

0.16%

0.699

Bacillales: Planococcaceae

0.07%

0.44%

0.699

0.11%

0.301

Erysipelotrichales: Erysipelatoclostridiaceae

0.09%

0.86%

0.008*

0.37%

0.014*

Erysipelotrichales: Erysipelotrichaceae

1.36%

2.09%

0.087

0.32%

0.008*

Lactobacillales: Enterococcaceae

2.09%

2.19%

0.059

0.40%

0.038*

Lactobacillales: Lactobacillaceae

17.35%

10.55%

0.125

14.87%

0.301

RF39: RF39

0.31%

1.05%

0.301

0.68%

0.087

Staphylococcales: Staphylococcaceae

1.12%

0.65%

0.458

0.02%

0.014*

Christensenellales: Christensenellaceae

1.00%

0.25%

0.087

1.50%

0.301

Clostridia_UCG-014: Clostridia_UCG-014

6.72%

10.71%

0.087

3.94%

0.059

Clostridia_vadinBB60_group:

Clostridia_vadinBB60_group

0.31%

0.54%

0.059

0.47%

0.232

Clostridiales: Clostridiaceae

0.51%

0.85%

0.174

0.08%

0.232

Lachnospirales: Lachnospiraceae

10.06%

8.77%

0.301

18.47%

0.038*

Monoglobales: Monoglobaceae

0.13%

0.08%

0.301

0.20%

0.174

Oscillospirales: Butyricicoccaceae

0.08%

0.07%

0.301

0.23%

0.005*

Oscillospirales: Oscillospiraceae

5.80%

7.89%

0.125

11.17%

0.038*

Oscillospirales: Ruminococcaceae

6.36%

9.04%

0.038*

13.38%

0.008*

Oscillospirales: UCG-010

0.45%

0.59%

0.377

0.51%

0.301

Oscillospirales: [Eubacterium]_

coprostanoligenes_group

0.43%

1.18%

0.174

1.31%

0.008*

Peptococcales: Peptococcaceae

0.02%

0.03%

0.125

0.16%

0.005*

Peptostreptococcales

-Tissierellales: Anaerovoracaceae

0.87%

0.78%

0.301

0.43%

0.232

Peptostreptococcales

-Tissierellales: Peptostreptococcaceae

1.88%

1.57%

0.301

0.46%

0.024*

Saccharimonadales: Saccharimonadaceae

0.16%

0.12%

0.232

0.01%

0.005*

Enterobacterales: Enterobacteriaceae

0.73%

1.45%

0.087

0.02%

0.059

Enterobacterales: Morganellaceae

15.10%

4.68%

0.087

1.12%

0.059

Verrucomicrobiales: Akkermansiaceae

1.26%

0.04%

0.174

2.38%

0.087

**Family**

**Ag**

**SiO_2_**

VC: vehicle-treated controls, Ag: Ag NP-treated animals, SiO_2_: SiO_2_ NP-treated animals. Asterisks mark significant changes, *p*-values were obtained by using the Mann-Whitney-U-test, N = 5 for each treatment group.

**Table S11: Relative abundance of bacterial genera (**≥ **1% in at least one group) in the gut microbiota of male Wistar rats**

**VC**

**mean**

**mean**

**p-value**

**mean**

**p-value**

Bacteroidaceae:

*Bacteroides*

4.60%

6.85%

0.087

6.58%

0.059

Muribaculaceae:

*Muribaculaceae*

14.31%

15.13%

0.377

11.80%

0.087

Erysipelotrichaceae:

*Turicibacter*

1.11%

1.84%

0.087

0.15%

0.008*

Enterococcaceae:

*Enterococcus*

2.09%

2.19%

0.059

0.40%

0.038*

Lactobacillaceae:

*Lactobacillus*

17.35%

10.54%

0.125

14.86%

0.301

RF39: RF

39

0.31%

1.05%

0.301

0.68%

0.087

Staphylococcaceae:

*Staphylococcus*

1.12%

0.65%

0.458

0.02%

0.014*

Christensenellaceae:

*Christensenellaceae*

0.98%

0.22%

0.059

1.47%

0.301

Clostridia_UCG-014:

*Clostridia_UCG*

-

014

6.72%

10.71%

0.087

3.94%

0.059

Lachnospiraceae:

*Lachnospiraceae*

*_NK4A136_group*

2.06%

0.87%

0.458

2.84%

0.232

Oscillospiraceae:

*NK4A214_group*

1.22%

1.46%

0.301

1.42%

0.232

Oscillospiraceae:

*UCG-005*

0.67%

1.85%

0.038*

1.10%

0.087

Ruminococcaceae:

*Incertae_Sedis*

1.40%

1.73%

0.458

0.77%

0.059

Ruminococcaceae:

*Ruminococcus*

0.66%

0.57%

0.377

1.32%

0.087

Ruminococcaceae:

*[Eubacterium]_siraeum_group*

0.26%

0.23%

0.125

2.42%

0.008*

[Eubacterium]_coprostanoligenes_group:

*[Eubacterium]_*

*coprostanoligenes_group*

0.43%

1.18%

0.174

1.31%

0.008*

Peptostreptococcaceae:

*Romboutsia*

1.86%

1.55%

0.301

0.46%

0.024*

Morganellaceae:

*Morganella*

1.53%

1.29%

0.301

0.20%

0.059

Morganellaceae:

*Proteus*

13.55%

3.39%

0.087

0.92%

0.059

Akkermansiaceae:

*Akkermansia*

1.26%

0.04%

0.174

2.38%

0.087

**Genus**

**Ag**

**SiO_2_**

VC: vehicle-treated controls, Ag: Ag NP-treated animals, SiO_2_: SiO_2_ NP-treated animals. Mean values expressed as % of the total gut microbiota, N = 5 for each treatment group, asterisks mark significant changes, *p*-values were obtained by using the Mann-Whitney-U-test.

**Table S12: Individual sample and median values for the relative abundance of selected most abundant bacterial genera (**≥ **1% in at least one group) in the gut microbiota of male Wistar rats**

VC: vehicle-treated controls, Ag50: Ag NP-treated animals, SiO_2_: SiO_2_ NP-treated animals.

Median values expressed as % of the total gut microbiota, N = 5 for each treatment group. Sample numbers 1 to 5 refer to the individual animals of each test group, respectively. These data are displayed as scatter plots in Fig. 2 of the Results and Discussion section.

**Table S13: Overall comparison of plasma metabolite changes in male Wistar rats after 28 days of treatment with Ag NP or SiO_2_ NP and changes observed after treatments with different antibiotics.**

Oral treatment with SiO_2_ nanoparticles (SiO_2_ naked), or Ag nanoparticles (Ag50 EO) or different antibiotics as indicated. Statistically significant changes (Welch test; p < 0.2) are coloured in red, representing increase or yellow, representing decrease, N = 5. In parts, the data shown for the different antibiotics were already published ^[4, 5, 6, 7]^.

**Table S14: Physicochemical characterization of the test substances (adapted from: Hellack *et al.,* 2012; Wohlleben *et al.,* 2013 [8, 9]).**

| **Property** | **Method** | **Units** | **SiO_2_** | **Ag** |
| --- | --- | --- | --- | --- |
| Form of delivery |  | weight% | Suspension (40%) | Suspension (25%) |
| Intended surface functionalisation | Educts in synthesis | Qualitative | None | EO |
| Particle morphology | LM / SEM | Qualitative | uniform, globular | irregular, globular |
| Crystallinity | XRD | Qualitative | amorphous | cubic, face-centered |
| Primary particle size (number mean) | TEM | nm | 15 | 7 |
| Primary particle size distribution | TEM | nm | 5 – 50 | 2 – 85 |
| Particle size (H_2_O) | DLS D_50_ (H_2_O) | nm | 40 | 40 |
| Particle size (mass) / dispersability (H_2_O) | AUC D_50_ (H_2_O) / AAN | nm / qualitative | 19 / 1 | 34 / 1 |
| Particle size (mass) / dispersability in DMEM/FCS | AUC  (D_50_ / AAN) | nm / qualitative | 420 / 28 | 38 / 1 |
| Particle size (mass) / dispersability in PBS+BSA (pH value: 7.4) | AUC (NP diameter / non-adsorbed albumin) | nm / % | 15 / non-ads. albumin: 42% | not determined |
| Surface composition | XPS /  supported by SIMS | Atom-% /  qualitative | Si: 29; O: 66; C: 4  (C-C, C-H, C-O, C=O); Na: 1 | C: 63; O: 23.5; Ag: 13.5 / impurities: organic C_x_H_y_O_2_ |
| Iso-electric point | Electrophoretic mobility titration | pH | <1 | 2.5 |
| Zeta-potential at pH 7.4 | Zetasizer | mV | -39 | -20 |
| Surface reactivity * | ESR / CPH spin trap | Relative to D_2_O | 4 / p-f s: 0.88 | not available |
| Formation of OH radicals [10] * | ESR / DMPO spin trap | Relative to D_2_O | 11 / p-f s: 6.3 | not available |

*: Surface reactivity and formation of reactive oxygen species ^[10]^ were determined relative to the ‘reference material’ deuterium oxide (D_2_O; ^2^H_2_O). Assuming a 30% variability of the methodology, only measurements >1.3 are considered relevant. Of note, this value should only serve as a guiding principle, and not as an absolute value.

AAN: average agglomeration number; derived from the ratio of the volume-based median particle size to the average equivalent spherical volume derived from the BET gas adsorption.

ROS: reactive oxygen species, p-f s: particle-free supernatant.

**Table S15: Altered plasma metabolite pathways in Ag NP or SiO_2_ NP-treated rats and their functional relationship with the gut microbiota**.

| **Metabolite** | **Trend SiO_2_** | **Trend Ag** | **Related Pathways** | **Source** | **Microbially mediated conversions** | **Impact of the gut microbiome** |
| --- | --- | --- | --- | --- | --- | --- |
|  |  |  |  |  |  |  |
| **Phenylalanine (Phe)** | ~ | **↓*** | Phenylalanine & Tyrosine metabolism | dietary and microbial (e.g. *Morganella*, *Bacillus, Echerichia,^[11]^*) | Phenylacetic acid, *P*-cresol, Phenylethylamine (PEA), e.g. by *Morganella morganii* ^[11]^ | Triggering of host physiological responses, Fine-tuning of host physiology via Production of Phe, agonist for G-protein coupled receptors, and PEA, a dopamine receptor antagonist [11] |
| **Proline (Pro)** | ~ | **↑*** | Arginine and Proline metabolism | dietary from L-glutamate, microbial from 1-Pyrroline-5 carboxylic acid (P5C)^[12]^ | Metabolization of Hyp, a derivative of Pro (e.g. *Clostridium*)[12] | Hyp metabolism may affect Pro availability of the host and may impact host stress and apoptosis ^[12, 13]^ |
| **Tryptophan (Trp)** | ~ | **↑*** | Serotonine pathway, Kynurenine pathway, Indole pathway | from dietary proteins | Production of Indole derivatives (e.g. IAA, IAld, IA) by various genera, e.g. *Akkermansia, Lactobacillus, Peptostreptococus, Eubacterium, Bifidobacterium* ^[14, 15, 16]^ | Host gut metabolic interaction, maintenance of Trp homeostasis (altered Trp homeostasis associated with neurologic and psychologic disorders and chronic inflammation ^[17]^ |
| **Indole-3-acetic acid*** | **↓*** | **↓*** | Indole pathway | 100% microbiota-derived from dietary Tryptophan via Indole-3 acetamide by various genera, e.g. *Akkermansia, Lactobacillus, Peptostreptococus, Eubacterium, Bifidobacterium* | Reduction to Indole-3 aldehyde (IAld), ligand of the arylhydrocarbon receptor (AHR) ^[14, 18]^ | Driving mucosal immune responses, maintenance of intestinal homeostasis, epithelial surveillance, intraepithelial lymphocyte maintenance ^[14, 18, 19]^ |
| **trans-4-Hydroxyproline (Hyp)** | **↓**** | ~ | Hyp metabolism | Hydroxylation of Proline (posttranlational modification) | Metabolization of Hyp to P5C and Proline, chemical reversion of Proline hydroxylation (e.g. *Clostridium* ^[12]^ | Hyp metabolism may affect Pro availability of the host and may impact host stress and apoptosis [12, 13] |
| **Hexoses-2** | **↑*** | **↑*** | Carbohydrate metabolism | dietary from plant fibers, microbial from pentoses via pentose phosphate pathway (*Prevotella copri*) [20] | Fermentation to Lactate, Succinate or SCFAs (Acetate, Propionate, Butyrate), Hydrogen and Carbondioxide ^[21]^ | Provisioning of energy and carbon source to the host ^[20]^ |
| **dihomo-γ-Linolenic acid (C20:cis[8,11,14]3) (DGLA)** | ~ | **↑*** | γ-Linoleic acid metabolism | Elongation product of dietary γ-Linoleic acid mediated by host and gut microbiota | Elongation of γ-Linoleic acid to dihomo-γ-Linoleic acid ^[22]^ | Co-Production of DGLA, precursor of arachidonic acid (inflammatory) and bioactive eicosanoids (anti-inflammatory) ^[23]^, Interaction with host lipid homeostasis [22] |
| **Phosphatidylcholine No 02 (PC)** | **↓*** | **↓'** | Phosphatidylcholine-Choline metabolic pathway | dietary | Breakdown of PC to trimethyl-amine (TMA), which is oxidized by hepatic flavin-containing mono-oxygenases to TMAO ^[24]^ | Gut microbiota metabolism of PC promotes cardiovascular disease via TMAO, a cardiovascular risk factor ^[24, 25]^ |
| **Sphingomyelin (d18:2,C18:0) (SM)** | **↓'** | **↓*** | Sphingolipid Metabolic pathway | dietary or synthesis via ER-produced Ceramide and transfer of phosphocholine from PC [26], microbial, e.g. Bacteroides [27] | SM from *Bacteroides* form membrane functional units for signaling ^[27]^ | SM has protective properties against gut dysbiosis and inflammation ^[28]^, positive impact on bacterial survival under stress ^[27]^ |
| **Malate** | **↓*** | ~ | Energy metabolism | dietary, from Fumarate (TCA cycle), Oxalacetate (Malate-Aspartate shuttle) | Synthesis of Propionate (e.g. Bacteroidetes) or Succinate (e.g. *Prevotella* and *Ruminococcus*) via Succinate pathway ^[21, 29]^ | Energy supply, Impact on lipogenesis and carcinogenesis ^[30]^ |
| **3,4-Dihydroxyphenylglycol (DOPEG)** | ~ | **↓**** | Catecholamine metabolism | Deamination of norepinephrine and epinephrine produces a reactive aldehyde that is reduced to form DHPG ^[31]^ | Production of norepinephrine (e.g. *Proteus vulgaris*) ^[32]^ | Modulation of neurotransmission, communication along the gut-brain axis ^[32]^ |
| **4-Hydroxy-3-methoxyphenylglycol (HMPG)** | **↓'** | **↓*** | Catecholamine metabolism | Intermediate aldehyde of norepinephrine metabolism is reduced to HMPG ^[33]^ | Production of norepinephrine (e.g. *Proteus vulgaris*) ^[32]^ | Modulation of neurotransmission, communication along the gut-brain axis ^[32]^ |
| **Epinephrine** | **↓*** | ~ | Catecholamine metabolism | produced from norepinephrine via Phenylethanolamine N-Methyltransferase | Production of norepinephrine (e.g. *Proteus vulgaris*) ^[32]^ | Modulation of neurotransmission, communication along the gut-brain axis ^[32]^ |
| **Metanephrine** | **↓*** | ~ | Catecholamine metabolism | produced from Epinephrine via catechol-o-methyltransferase | Production of norepinephrine (e.g. *Proteus vulgaris*) ^[32]^ | Modulation of neurotransmission, communication along the gut-brain axis ^[32]^ |
| **Allantoin** | ~ | **↑*** | Purine Catabolism | degradation product of uric acid | unknown | unknown |
| **Pseudouridine** | **↑*** | ~ | Posttranscriptional Modification (Pseudourinylation) | Isomerization of uridines, widely distributed in naturally occuring RNAs ^[34]^ | unknown | unknown |
| **Pantothenic acid** | **↓*** | ~ | Biosynthesis of Pantothenic acid pathway | dietary, microbial *de novo* synthesis from 2-Dihydro-pantoate and ß-Alanine (100% of the phylum Bacterioidetes, 95% of the phylum Proteobacteria) ^[35]^ | host-gut microbiota Co-production of Coenzyme A, intestinal conversion of Coenzyme A to pantothenic acid (Proteobacteria*,* Bacterioidetes) ^[35, 36]^ | impact on cell metabolism, growth, cell differentiation ^[36]^ |
| **Threonic acid** | **↓*** | ~ | Carbohydrate metabolism | dietary, microbial, degradation product of ascorbic acid ^[37]^ | host-gut microbiota co-production of threonic acid ^[37]^ | maintenance of gut barrier integrity, contribution to energy metabolism, Protection from intestinal inflammation ^[38]^ |
|  |  |  |  |  |  |  |
| ↑: upregulated, ↓: downregulated, ~: not significantly altered; ': *p* < 0.2, *: *p* < 0.05, **: *p* < 0.01, N=5 | | | | | | |

**Supplementary Figures**

**a**

**b**

**Fig. S1 Alpha diversity of the gut microbiota in male Wistar rats before (untreated) and after exposure to vehicle, Ag NP (Ag50) or SiO_2_NP (SiO_2_), shown as Shannon-Wiener index (a) or Inverse Simpson index (b).** UC: untreated control, VC: vehicle control, Ag50: Ag NP-treated rats, SiO_2_: SiO_2_ NP-treated rats. Values are means ± SD, N = 5 for treatment groups. Alpha diversity was calculated ASV-based and evaluated by Student‘s *t*-test No significant differences were noted between nanoparticle-treated rats and the vehicle controls (*p* < 0.05).


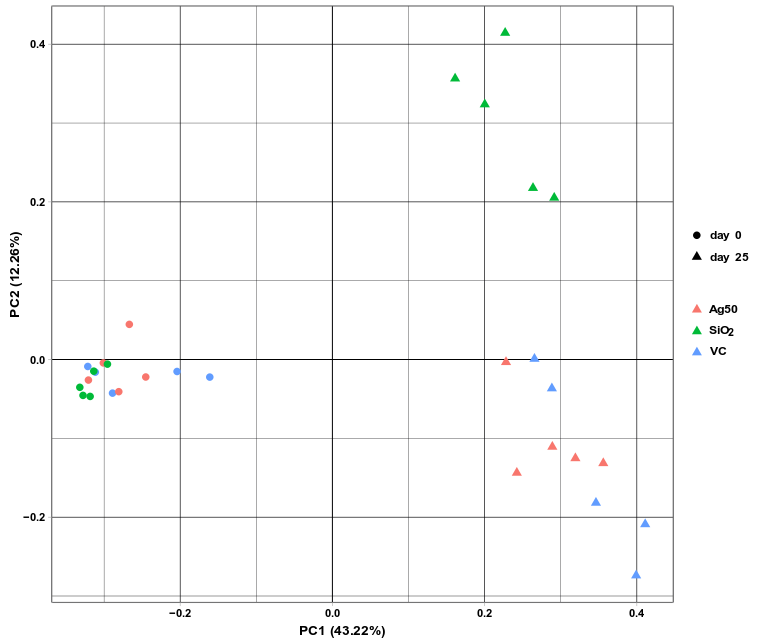


**a**


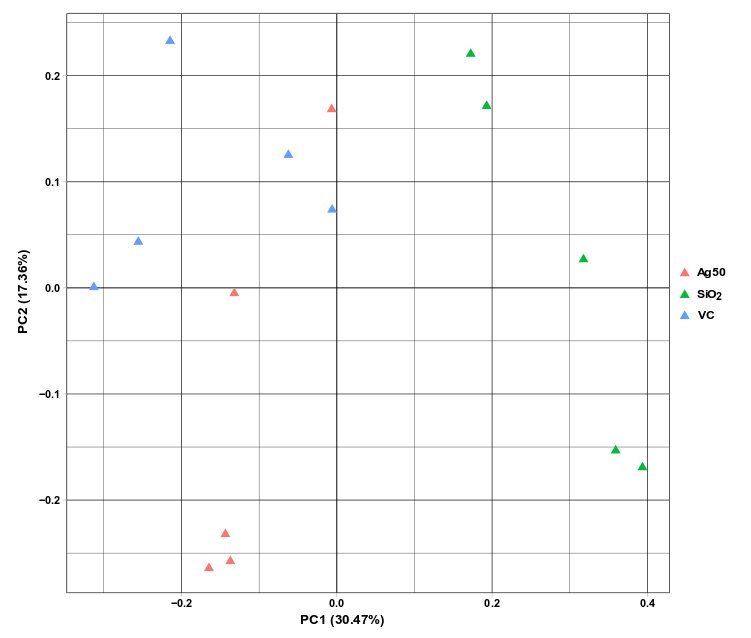


**b**

**Fig. S2 Beta-diversity of gut microbiota visualized as PCoA plots**. Beta-diversity illustrated for samples of untreated male Wistar rats (at day 0) compared to the same animals after 25 days of gavage treatment with vehicle, Ag NP or SiO_2_ NP (a), or samples from male Wistar rats after 25 days of gavage treatment only to compare treatments with vehicle, Ag NP and SiO_2_ NP (b). VC: vehicle control, Ag50: Ag NP, SiO_2_: SiO_2_ NP. Bray-Curtis distance values were calculated using ASV data of the indicated samples, collected at day 0 and/or at day 25 after treatment.

**a**

**b**


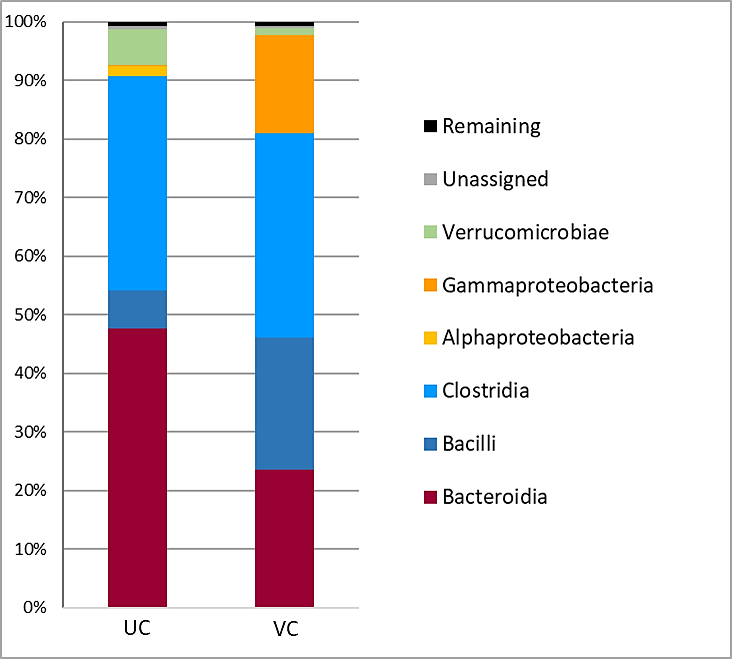


**Fig. S3** **Relative abundance of bacterial phyla (a) and classes (b) in the gut microbiota (**≥ **1%) of male Wistar rats before (untreated) and after a 25-day gavage of PBS + BSA (vehicle).** UC: Untreated control, mean values of the gut microbiota composition in feces collected at day 0 before the beginning of the experiment, VC: Vehicle control, mean values of the gut microbiota composition from feces of five animals after gavage with the vehicle PBS + BSA, collected at day 25.

**Fig. S4 Scatter plots obtained for selected most abundant genera after exposure to either Ag or SiO_2_ NP** (median relative abundance was ≥ 1% in at least one group). VC: vehicle control; Ag50: Ag NP; SiO_2_: SiO_2_ NP, N = 5, *p*-values were obtained by using the Mann-Whitney-U-test.

**
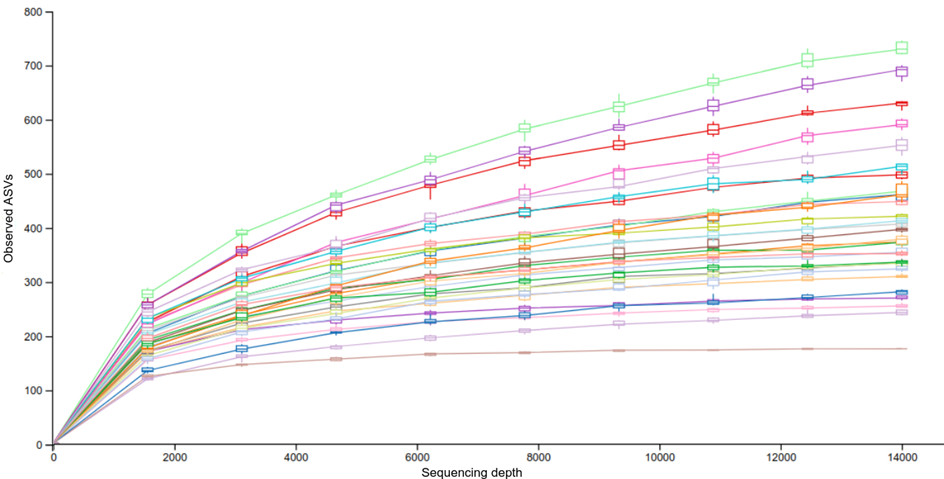
**

**Fig. S5** **Rarefaction curves of all animals used for the analyses** (samples of untreated rats at day 0; vehicle controls, Ag NP and SiO_2_NP-treated samples at day 25, respectively). Different colors display the different individual samples. The box plots represent the distribution of the observed ASVs for each sample at each even sampling depth.

**References**

1. Han H, Xiao H, Zhang K, Lu Z. Impact of 4-epi-oxytetracycline on the gut microbiota and blood metabolomics of Wistar rats. Sci Rep. 2016;6:23141; doi: 10.1038/srep23141. <http://www.ncbi.nlm.nih.gov/pubmed/26976662>.

2. Mariat D, Firmesse O, Levenez F, Guimaraes V, Sokol H, Dore J, et al. The Firmicutes/Bacteroidetes ratio of the human microbiota changes with age. BMC Microbiol. 2009;9:123; doi: 10.1186/1471-2180-9-123. <http://www.ncbi.nlm.nih.gov/pubmed/19508720>.

3. Buesen R, Landsiedel R, Sauer UG, Wohlleben W, Groeters S, Strauss V, et al. Effects of SiO(2), ZrO(2), and BaSO(4) nanomaterials with or without surface functionalization upon 28-day oral exposure to rats. Arch Toxicol. 2014;88 10:1881-906; doi: 10.1007/s00204-014-1337-0. <http://www.ncbi.nlm.nih.gov/pubmed/25164825>.

4. Behr C, Kamp H, Fabian E, Krennrich G, Mellert W, Peter E, et al. Gut microbiome-related metabolic changes in plasma of antibiotic-treated rats. Arch Toxicol. 2017;91 10:3439-54; doi: 10.1007/s00204-017-1949-2. <https://www.ncbi.nlm.nih.gov/pubmed/28337503>.

5. Behr C, Ramirez-Hincapie S, Cameron HJ, Strauss V, Walk T, Herold M, et al. Impact of lincosamides antibiotics on the composition of the rat gut microbiota and the metabolite profile of plasma and feces. Toxicol Lett. 2018;296:139-51; doi: 10.1016/j.toxlet.2018.08.002. <https://www.ncbi.nlm.nih.gov/pubmed/30102961>.

6. Behr C, Slopianka M, Haake V, Strauss V, Sperber S, Kamp H, et al. Analysis of metabolome changes in the bile acid pool in feces and plasma of antibiotic-treated rats. Toxicol Appl Pharmacol. 2019;363:79-87; doi: 10.1016/j.taap.2018.11.012. <https://www.ncbi.nlm.nih.gov/pubmed/30502395>.

7. Behr C, Sperber S, Jiang X, Strauss V, Kamp H, Walk T, et al. Microbiome-related metabolite changes in gut tissue, cecum content and feces of rats treated with antibiotics. Toxicol Appl Pharmacol. 2018;355:198-210; doi: 10.1016/j.taap.2018.06.028. <https://www.ncbi.nlm.nih.gov/pubmed/30008377>.

8. Hellack B, Hülser T, Izak E, Kuhlbusch T, Meyer F, Spree M, et al: Characterization report for all nanoGEM materials. 2012.

9. Wohlleben W, Ma-Hock L, Boyko V, Cox G, Egenolf H, Freiberger H, et al. Nanospecific Guidance in REACH: A Comparative Physical-Chemical Characterization of 15 Materials with Methodical Correlations. J Ceram Sci Technol. 2013;4 2:93-104; doi: 10.4416/Jcst2012-00045. <Go to ISI>://WOS:000209199600006.

10. Scher JU, Sczesnak A, Longman RS, Segata N, Ubeda C, Bielski C, et al. Expansion of intestinal Prevotella copri correlates with enhanced susceptibility to arthritis. Elife. 2013;2:e01202; doi: 10.7554/eLife.01202. <http://www.ncbi.nlm.nih.gov/pubmed/24192039>.

11. Pandey S, Maharana J, Shukla AK. The Gut Feeling: GPCRs Enlighten the Way. Cell Host Microbe. 2019;26 2:160-2; doi: 10.1016/j.chom.2019.07.018. <https://www.ncbi.nlm.nih.gov/pubmed/31415748>.

12. Levin BJ, Huang YY, Peck SC, Wei Y, Martinez-Del Campo A, Marks JA, et al. A prominent glycyl radical enzyme in human gut microbiomes metabolizes trans-4-hydroxy-l-proline. Science. 2017;355 6325; doi: 10.1126/science.aai8386. <https://www.ncbi.nlm.nih.gov/pubmed/28183913>.

13. Phang JM, Liu W, Zabirnyk O. Proline metabolism and microenvironmental stress. Annu Rev Nutr. 2010;30:441-63; doi: 10.1146/annurev.nutr.012809.104638. <https://www.ncbi.nlm.nih.gov/pubmed/20415579>.

14. Roager HM, Licht TR. Microbial tryptophan catabolites in health and disease. Nat Commun. 2018;9 1:3294; doi: 10.1038/s41467-018-05470-4. <https://www.ncbi.nlm.nih.gov/pubmed/30120222>.

15. Lamas B, Richard ML, Leducq V, Pham HP, Michel ML, Da Costa G, et al. CARD9 impacts colitis by altering gut microbiota metabolism of tryptophan into aryl hydrocarbon receptor ligands. Nat Med. 2016;22 6:598-605; doi: 10.1038/nm.4102. <https://www.ncbi.nlm.nih.gov/pubmed/27158904>.

16. van Beek AA, Hugenholtz F, Meijer B, Sovran B, Perdijk O, Vermeij WP, et al. Frontline Science: Tryptophan restriction arrests B cell development and enhances microbial diversity in WT and prematurely aging Ercc1(-/Delta7) mice. J Leukoc Biol. 2017;101 4:811-21; doi: 10.1189/jlb.1HI0216-062RR. <https://www.ncbi.nlm.nih.gov/pubmed/27418353>.

17. Comai S, Bertazzo A, Brughera M, Crotti S. Tryptophan in health and disease. Adv Clin Chem. 2020;95:165-218; doi: 10.1016/bs.acc.2019.08.005. <https://www.ncbi.nlm.nih.gov/pubmed/32122523>.

18. Lamas B, Martins Breyner N, Houdeau E. Impacts of foodborne inorganic nanoparticles on the gut microbiota-immune axis: potential consequences for host health. Part Fibre Toxicol. 2020;17 1:19; doi: 10.1186/s12989-020-00349-z. <https://www.ncbi.nlm.nih.gov/pubmed/32487227>.

19. Lavelle A, Sokol H. Gut microbiota-derived metabolites as key actors in inflammatory bowel disease. Nat Rev Gastroenterol Hepatol. 2020;17 4:223-37; doi: 10.1038/s41575-019-0258-z. <https://www.ncbi.nlm.nih.gov/pubmed/32076145>.

20. Basen M, Kurrer SE. A close look at pentose metabolism of gut bacteria. FEBS J. 2021;288 6:1804-8; doi: 10.1111/febs.15575. <https://www.ncbi.nlm.nih.gov/pubmed/33063458>.

21. Reichardt N, Duncan SH, Young P, Belenguer A, McWilliam Leitch C, Scott KP, et al. Phylogenetic distribution of three pathways for propionate production within the human gut microbiota. ISME J. 2014;8 6:1323-35; doi: 10.1038/ismej.2014.14. <https://www.ncbi.nlm.nih.gov/pubmed/24553467>.

22. Kindt A, Liebisch G, Clavel T, Haller D, Hormannsperger G, Yoon H, et al. The gut microbiota promotes hepatic fatty acid desaturation and elongation in mice. Nat Commun. 2018;9 1:3760; doi: 10.1038/s41467-018-05767-4. <https://www.ncbi.nlm.nih.gov/pubmed/30218046>.

23. Whelan J. The health implications of changing linoleic acid intakes. Prostaglandins Leukot Essent Fatty Acids. 2008;79 3-5:165-7; doi: 10.1016/j.plefa.2008.09.013. <https://www.ncbi.nlm.nih.gov/pubmed/18990554>.

24. Stremmel W, Schmidt KV, Schuhmann V, Kratzer F, Garbade SF, Langhans CD, et al. Blood Trimethylamine-N-Oxide Originates from Microbiota Mediated Breakdown of Phosphatidylcholine and Absorption from Small Intestine. PLoS One. 2017;12 1:e0170742; doi: 10.1371/journal.pone.0170742. <https://www.ncbi.nlm.nih.gov/pubmed/28129384>.

25. Wang Z, Klipfell E, Bennett BJ, Koeth R, Levison BS, Dugar B, et al. Gut flora metabolism of phosphatidylcholine promotes cardiovascular disease. Nature. 2011;472 7341:57-63; doi: 10.1038/nature09922. <https://www.ncbi.nlm.nih.gov/pubmed/21475195>.

26. Pralhada Rao R, Vaidyanathan N, Rengasamy M, Mammen Oommen A, Somaiya N, Jagannath MR. Sphingolipid metabolic pathway: an overview of major roles played in human diseases. J Lipids. 2013;2013:178910; doi: 10.1155/2013/178910. <https://www.ncbi.nlm.nih.gov/pubmed/23984075>.

27. An D, Na C, Bielawski J, Hannun YA, Kasper DL. Membrane sphingolipids as essential molecular signals for Bacteroides survival in the intestine. Proc Natl Acad Sci U S A. 2011;108 Suppl 1:4666-71; doi: 10.1073/pnas.1001501107. <https://www.ncbi.nlm.nih.gov/pubmed/20855611>.

28. Norris GH, Milard M, Michalski MC, Blesso CN. Protective properties of milk sphingomyelin against dysfunctional lipid metabolism, gut dysbiosis, and inflammation. J Nutr Biochem. 2019;73:108224; doi: 10.1016/j.jnutbio.2019.108224. <https://www.ncbi.nlm.nih.gov/pubmed/31654895>.

29. Louis P, Flint HJ. Formation of propionate and butyrate by the human colonic microbiota. Environ Microbiol. 2017;19 1:29-41; doi: 10.1111/1462-2920.13589. <https://www.ncbi.nlm.nih.gov/pubmed/27928878>.

30. Hosseini E, Grootaert C, Verstraete W, Van de Wiele T. Propionate as a health-promoting microbial metabolite in the human gut. Nutr Rev. 2011;69 5:245-58; doi: 10.1111/j.1753-4887.2011.00388.x. <https://www.ncbi.nlm.nih.gov/pubmed/21521227>.

31. Eisenhofer G, Kopin IJ, Goldstein DS. Catecholamine metabolism: a contemporary view with implications for physiology and medicine. Pharmacol Rev. 2004;56 3:331-49; doi: 10.1124/pr.56.3.1. <https://www.ncbi.nlm.nih.gov/pubmed/15317907>.

32. Strandwitz P. Neurotransmitter modulation by the gut microbiota. Brain Res. 2018;1693 Pt B:128-33; doi: 10.1016/j.brainres.2018.03.015. <https://www.ncbi.nlm.nih.gov/pubmed/29903615>.

33. Mardh G, Dingley AL, Auld DS, Vallee BL. Human class II (pi) alcohol dehydrogenase has a redox-specific function in norepinephrine metabolism. Proc Natl Acad Sci U S A. 1986;83 23:8908-12; doi: 10.1073/pnas.83.23.8908. <https://www.ncbi.nlm.nih.gov/pubmed/3466164>.

34. Charette M, Gray MW. Pseudouridine in RNA: what, where, how, and why. IUBMB Life. 2000;49 5:341-51; doi: 10.1080/152165400410182. <https://www.ncbi.nlm.nih.gov/pubmed/10902565>.

35. Magnusdottir S, Ravcheev D, de Crecy-Lagard V, Thiele I. Systematic genome assessment of B-vitamin biosynthesis suggests co-operation among gut microbes. Front Genet. 2015;6:148; doi: 10.3389/fgene.2015.00148. <https://www.ncbi.nlm.nih.gov/pubmed/25941533>.

36. Gominak SC. Vitamin D deficiency changes the intestinal microbiome reducing B vitamin production in the gut. The resulting lack of pantothenic acid adversely affects the immune system, producing a "pro-inflammatory" state associated with atherosclerosis and autoimmunity. Med Hypotheses. 2016;94:103-7; doi: 10.1016/j.mehy.2016.07.007. <https://www.ncbi.nlm.nih.gov/pubmed/27515213>.

37. Zheng X, Xie G, Zhao A, Zhao L, Yao C, Chiu NH, et al. The footprints of gut microbial-mammalian co-metabolism. J Proteome Res. 2011;10 12:5512-22; doi: 10.1021/pr2007945. <https://www.ncbi.nlm.nih.gov/pubmed/21970572>.

38. Sun L, Jia H, Li J, Yu M, Yang Y, Tian D, et al. Cecal Gut Microbiota and Metabolites Might Contribute to the Severity of Acute Myocardial Ischemia by Impacting the Intestinal Permeability, Oxidative Stress, and Energy Metabolism. Front Microbiol. 2019;10:1745; doi: 10.3389/fmicb.2019.01745. <https://www.ncbi.nlm.nih.gov/pubmed/31428065>.
